# Schizophrenia risk conferred by rare protein-truncating variants is conserved across diverse human populations

**DOI:** 10.1038/s41588-023-01305-1

**Published:** 2023-03-13

**Authors:** Dongjing Liu, Dara Meyer, Brian Fennessy, Claudia Feng, Esther Cheng, Jessica S. Johnson, You Jeong Park, Marysia-Kolbe Rieder, Steven Ascolillo, Agathe de Pins, Amanda Dobbyn, Dannielle Lebovitch, Emily Moya, Tan-Hoang Nguyen, Lillian Wilkins, Arsalan Hassan, Psychiatric Genomics Consortium Phase 3 Targeted Sequencing of Schizophrenia Study Team, Henry S. Aghanwa, Moin Ansari, Aftab Asif, Rubina Aslam, Jose L. Ayuso, Tim Bigdeli, Stefano Bignotti, Julio Bobes, Bekh Bradley, Peter Buckley, Murray J. Cairns, Stanley V. Catts, Abdul Rashid Chaudhry, David Cohen, Brett L. Collins, Angèle Consoli, Javier Costas, Benedicto Crespo-Facorro, Nikolaos P. Daskalakis, Michael Davidson, Kenneth L. Davis, Faith Dickerson, Imtiaz A. Dogar, Elodie Drapeau, Lourdes Fañanás, Ayman Fanous, Warda Fatima, Mar Fatjo, Cheryl Filippich, Joseph Friedman, John F. Fullard, Penelope Georgakopoulos, Marianna Giannitelli, Ina Giegling, Melissa J. Green, Olivier Guillin, Blanca Gutierrez, Herlina Y. Handoko, Stella Kim Hansen, Maryam Haroon, Vahram Haroutunian, Frans A. Henskens, Fahad Hussain, Assen V. Jablensky, Jamil Junejo, Brian J. Kelly, Shams-ud-Din A. Khan, Muhammad N. S. Khan, Anisuzzaman Khan, Hamid R. Khawaja, Bakht Khizar, Steven P. Kleopoulos, James Knowles, Bettina Konte, Agung A. A. A. Kusumawardhani, Naeemullah Leghari, Xudong Liu, Adriana Lori, Carmel M. Loughland, Khalid Mahmood, Saqib Mahmood, Dolores Malaspina, Danish Malik, Amy McNaughton, Patricia T. Michie, Vasiliki Michopolous, Esther Molina, María D. Molto, Asim Munir, Gerard Muntané, Farooq Naeem, Derek J. Nancarrow, Amina Nasar, Tanvir Nasr, Jude U. Ohaeri, Jurg Ott, Christos Pantelis, Sathish Periyasamy, Ana G. Pinto, Abigail Powers, Belén Ramos, Nusrat H. Rana, Mark Rapaport, Abraham Reichenberg, Safaa Saker-Delye, Ulrich Schall, Peter R. Schofield, Rodney J. Scott, Megan Shanahan, Cynthia Shannon Weickert, Calvin Sjaarda, Heather J. Smith, Jose Javier Suárez-Rama, Muhammad Tariq, Florence Thibaut, Paul A. Tooney, Muhammad Umar, Elisabet Vilella, Mark Weiser, Jin Qin Wu, Robert Yolken, Katherine E. Burdick, Joseph D. Buxbaum, Enrico Domenici, Sophia Frangou, Annette M. Hartmann, Claudine Laurent-Levinson, Dheeraj Malhotra, Carlos N. Pato, Michele T. Pato, Kerry Ressler, Panos Roussos, Dan Rujescu, Celso Arango, Alessandro Bertolino, Giuseppe Blasi, Luisella Bocchio-Chiavetto, Dominique Campion, Vaughan Carr, Janice M. Fullerton, Massimo Gennarelli, Javier González-Peñas, Douglas F. Levinson, Bryan Mowry, Vishwajit L. Nimgaokar, Giulio Pergola, Antonio Rampino, Jorge A. Cervilla, Margarita Rivera, Sibylle G. Schwab, Dieter B. Wildenauer, Mark Daly, Benjamin Neale, Tarjinder Singh, Michael C. O’Donovan, Michael J. Owen, James T. Walters, Muhammad Ayub, Anil K. Malhotra, Todd Lencz, Patrick F. Sullivan, Pamela Sklar, Eli A. Stahl, Laura M. Huckins, Alexander W. Charney

**Affiliations:** 1 Department of Genetics and Genomic Sciences, Icahn School of Medicine at Mount Sinai, New York, NY USA; 2 Wellcome Sanger Institute, Hinxton, UK; 3 Pamela Sklar Division of Psychiatric Genomics, Department of Genetics and Genomic Sciences, Icahn School of Medicine at Mount Sinai, New York, NY USA; 4 Department of Psychiatry, Icahn School of Medicine at Mount Sinai, New York, NY USA; 5 Virginia Institute for Psychiatric and Behavioral Genetics, Department of Psychiatry, Virginia Commonwealth University, Richmond, VA USA; 6 University of Peshawar, Peshawar, Pakistan; 7 Department of Psychiatry, Brigham and Women’s Hospital, Boston, MA USA; 8 Department of Psychiatry, Harvard Medical School, Boston, MA USA; 9 Centre for Computational and Systems Biology, Fondazione The Microsoft Research – University of Trento, Rovereto, Italy; 10 Department of Cellular, Computational and Integrative Biology, University of Trento, Trento, Italy; 11 Djavad Mowafaghian Centre for Brain Health, University of British Columbia, Vancouver, British Columbia Canada; 12 Department of Psychiatry and Psychotherapy, Medical University of Vienna, Vienna, Austria; 13 Faculté de Médecine Sorbonne Université, Groupe de Recherche Clinique n°15—Troubles Psychiatriques et Développement, Department of Child and Adolescent Psychiatry, Hôpital Universitaire de la Pitié-Salpêtrière, Paris, France; 14 Centre de Référence des Maladies Rares à Expression Psychiatrique, Department of Child and Adolescent Psychiatry, AP-HP Sorbonne Université, Hôpital Universitaire de la Pitié-Salpêtrière, Paris, France; 15 Department of Neuroscience and Rare Diseases, Roche Pharma Research and Early Development, F. Hoffmann-La Roche, Basel, Switzerland; 16 Department of Psychiatry and Behavioral Sciences, SUNY Downstate College of Medicine, New York, NY USA; 17 Division of Depression and Anxiety Disorders, McLean Hospital, Belmont, MA USA; 18 Mental Illness Research, Education, and Clinical Center (VISN 2 South), James J. Peters VA Medical Center, New York, NY USA; 19 Department of Child and Adolescent Psychiatry, Institute of Psychiatry and Mental Health, Hospital General Universitario Gregorio Marañón, Instituto de Investigación Sanitaria Gregorio Marañón, Madrid, Spain; 20 Centro de Investigación Biomédica en Red de Salud Mental, Madrid, Spain; 21 Department of Translational Biomedicine and Neuroscience, University of Bari Aldo Moro, Bari, Italy; 22 Department of Theoretical and Applied Sciences, eCampus University, Novedrate, Italy; 23 Genetics Unit, IRCCS Istituto Centro San Giovanni di Dio Fatebenefratelli, Brescia, Italy; 24 INSERM U1245, Rouen, France; 25 Centre Hospitalier du Rouvray, Rouen, France; 26 Neuroscience Research Australia, Sydney, New South Wales Australia; 27 School of Psychiatry, University of New South Wales, Sydney, New South Wales Australia; 28 Department of Psychiatry, School of Clinical Sciences, Monash University, Melbourne, Victoria Australia; 29 School of Medical Sciences, University of New South Wales, Sydney, New South Wales Australia; 30 Department of Molecular and Translational Medicine, University of Brescia, Brescia, Italy; 31 Department of Psychiatry, Stanford University, Stanford, CA USA; 32 Queensland Brain Institute, The University of Queensland, Brisbane, Queensland Australia; 33 Queensland Centre for Mental Health Research, The University of Queensland, Brisbane, Queensland Australia; 34 Department of Psychiatry, University of Pittsburgh School of Medicine, Western Psychiatric Hospital, Pittsburgh, PA USA; 35 Department of Human Genetics, Graduate School of Public Health, University of Pittsburgh, Pittsburgh, PA USA; 36 Institute of Neurosciences, Biomedical Research Centre, University of Granada, Granada, Spain; 37 Department of Psychiatry, San Cecilio University Hospital, University of Granada, Granada, Spain; 38 Department of Biochemistry and Molecular Biology II, Faculty of Pharmacy, University of Granada, Granada, Spain; 39 Molecular Horizons, Faculty of Science, Medicine and Health, University of Wollongong, Wollongong, New South Wales Australia; 40 The University of Western Australia, Perth, Western Australia Australia; 41 Analytic and Translational Genetics Unit, Department of Medicine, Massachusetts General Hospital, Boston, MA USA; 42 Stanley Center for Psychiatric Research, Broad Institute of MIT and Harvard, Cambridge, MA USA; 43 Program in Medical and Population Genetics, Broad Institute of MIT and Harvard, Cambridge, MA USA; 44 Institute for Molecular Medicine Finland, University of Helsinki, Helsinki, Finland; 45 MRC Centre for Neuropsychiatric Genetics and Genomics, Division of Psychological Medicine and Clinical Neurosciences, Cardiff University, Cardiff, UK; 46 University College London, London, UK; 47 Department of Psychiatry, Queen’s University, Kingston, Ontario Canada; 48 Department of Psychiatry, Zucker School of Medicine at Hofstra/Northwell, Hempstead, NY USA; 49 Institute for Behavioral Science, Feinstein Institutes for Medical Research, Manhasset, NY USA; 50 Division of Psychiatry Research, The Zucker Hillside Hospital, Northwell Health, New York, NY USA; 51 Departments of Genetics and Psychiatry, University of North Carolina, Chapel Hill, NC USA; 52 Department of Medical Epidemiology and Biostatistics, Karolinska Institutet, Stockholm, Sweden; 53 Regeneron Pharmaceuticals, Tarrytown, NY USA; 54 St Andrew’s Toowoomba Hospital, Toowoomba, Queensland Australia; 55 Sir Cowasjee Jehangir Institute of Psychiatry, Hyderabad, Pakistan; 56 King Edward Medical University, Lahore, Pakistan; 57 Allama Iqbal Medical College, Lahore, Pakistan; 58 Department of Psychiatry, Universidad Autónoma de Madrid, Madrid, Spain; 59 Hospital Universitario de La Princesa, Instituto de Investigación Sanitaria, Madrid, Spain; 60 SUNY Downstate Health Sciences University, Brooklyn, NY USA; 61 Psychiatry Unit, IRCCS Istituto Centro San Giovanni di Dio Fatebenefratelli, Brescia, Italy; 62 Faculty of Medicine and Health Sciences – Psychiatry, Universidad de Oviedo, Institute of Health Research of Principado de Asturias, Instituto de Neurociencias del Principado de Asturias, Oviedo, Spain; 63 Department of Psychiatry and Behavioral Sciences, Emory University, Atlanta, GA USA; 64 Virginia Commonwealth University, Richmond, VA USA; 65 School of Biomedical Sciences and Pharmacy, University of Newcastle, Newcastle, New South Wales Australia; 66 Hunter Medical Research Institute, Newcastle, New South Wales Australia; 67 Centre for Brain and Mental Health Research, The University of Newcastle, Newcastle, New South Wales Australia; 68 Brain and Mind Centre, The University of Sydney, Sydney, New South Wales Australia; 69 School of Medicine, The University of Queensland, Brisbane, Queensland Australia; 70 New Millat Brain Center, Sahiwal, Pakistan; 71 Institut des Systèmes Intelligents et de Robotique, CNRS UMR7222, Sorbonne Université, Campus Pierre et Marie Curie, Faculté des Sciences et Ingénierie, Paris, France; 72 Instituto de Investigación Sanitaria de Santiago de Compostela, Complexo Hospitalario Universitario de Santiago de Compostela, Servizo Galego de Saúde, Santiago de Compostela, Spain; 73 Hospital Universitario Virgen del Rocío, Department of Psychiatry, Universidad de Sevilla, Sevilla, Spain; 74 Harvard Medical School, Boston, MA USA; 75 McLean Hospital, Belmont, MA USA; 76 Nicosia University School of Medicine, Nicosia, Cyprus; 77 Icahn School of Medicine at Mount Sinai, New York, NY USA; 78 Sheppard Pratt Hospital, Baltimore, MD USA; 79 District Headquarter Hospital Failsalbad, Failsalbad, Pakistan; 80 Department of Evolutionary Biology, Ecology and Environmental Sciences, Faculty of Biology, University of Barcelona, Barcelona, Spain; 81 University of Arizona, Tuscon, AZ USA; 82 Veterans Affairs, New York, NY USA; 83 University of Punjab, Lahore, Pakistan; 84 FIDMAG Germanes Hospitalàries Research Foundation, Barcelona, Spain; 85 Departament de Biologia Evolutiva, Ecologia i Ciències Ambientals, Facultat de Biologia, Universitat de Barcelona, Barcelona, Spain; 86 UFR Santé, Université de Rouen Normandie, Rouen, France; 87 Department of Psychiatry, Faculty of Medicine, University of Granada, Granada, Spain; 88 Drug Discovery Group, Cell and Molecular Biology Department, Cancer Programme, QIMR Berghofer Medical Research Institute, Brisbane, Queensland Australia; 89 Professor Dr. Haroon Rashid Clinic, Lahore, Pakistan; 90 School of Medicine and Public Health, University of Newcastle, Newcastle, New South Wales Australia; 91 Lahore Institute of Research and Development, Lahore, Pakistan; 92 Centre for Clinical Research in Neuropsychiatry, The University of Western Australia, Perth, Western Australia Australia; 93 Department of Psychiatry and Behavioural Sciences, Liaquat University of Medical and Health Sciences, Jamshoro, Pakistan; 94 Al-Shamas Hospital, Sargodha, Pakistan; 95 Queen’s University, Kingston, Ontario Canada; 96 Nai Zindage Psychiatric Hospital, Multan, Pakistan; 97 Azad Jammu and Kashmir Medical College, Muzaffarabad, Pakistan; 98 Department of Psychiatry, Cipto Mangunkusumo General Hospital, Universitas Indonesia, Jakarta, Indonesia; 99 Nishtar Medical University, Multan, Pakistan; 100 School of Psychology, University of Newcastle, Newcastle, New South Wales Australia; 101 Ar-Rahma Hospital, Multan, Pakistan; 102 University of Health Sciences, Lahore, Pakistan; 103 Ameena Clinic, Gujranwala, Pakistan; 104 Department of Nursing, Faculty of Health Sciences, University of Granada, Granada, Spain; 105 Department of Genetics, University of Valencia, Valencia, Spain; 106 Wali Neuropsychiatric Centre, Faisalabad, Pakistan; 107 Hospital Universitari Institut Pere Mata, IISPV, Universitat Rovira i Virgili, Reus, Spain; 108 Center for Addiction and Mental Health, Toronto, Ontario Canada; 109 Department of Surgery, University of Michigan, Ann Arbor, MI USA; 110 Department of Psychological Medicine, University of Nigeria Teaching Hospital, Enugu, Nigeria; 111 Laboratory of Statistical Genetics, The Rockefeller University, New York, NY USA; 112 Melbourne Neuropsychiatry Centre, University of Melbourne and Melbourne Health, Melbourne, Victoria Australia; 113 The Florey Institute of Neuroscience and Mental Health, University of Melbourne, Parkville, Melbourne Australia; 114 NorthWestern Mental Health, Melbourne, Victoria Australia; 115 Bioaraba Health Research Institute, OSI Araba, University Hospital, University of the Basque Country, Vitoria, Spain; 116 Parc Sanitari Sant Joan de Déu, Barcelona, Spain; 117 Punjab Institute of Mental Health, Lahore, Pakistan; 118 University of Utah, Salt Lake City, UT USA; 119 James J. Peters VA Medical Center, New York, NY USA; 120 Généthon, Paris, France; 121 Priority Centre for Brain and Mental Health Research, The University of Newcastle, Mater Hospital, Newcastle, New South Wales Australia; 122 Division of Molecular Medicine, NSW Health Pathology North, Newcastle, New South Wales Australia; 123 Department of Neuroscience, SUNY Upstate Medical University, Syracuse, NY USA; 124 Grupo de Medicina Xenómica, Universidade de Santiago de Compostela, Santiago de Compostela, Spain; 125 Shafique Psychiatric Clinic, Peshawar, Pakistan; 126 Université de Paris, Faculté de Médecine, Hôpital Cochin-Tarnier, Paris, France; 127 INSERM U1266, Institut de Psychiatrie et de Neurosciences, Paris, France; 128 Sheba Medical Center, Ramat Gan, Israel; 129 School of Life and Environmental Sciences, University of Sydney, Sydney, New South Wales Australia; 130 Stanley Neurovirology Laboratory, Department of Pediatrics, Johns Hopkins School of Medicine, Baltimore, MD USA

**Keywords:** Schizophrenia, Genetic association study, Genetics research

## Abstract

Schizophrenia (SCZ) is a chronic mental illness and among the most debilitating conditions encountered in medical practice. A recent landmark SCZ study of the protein-coding regions of the genome identified a causal role for ten genes and a concentration of rare variant signals in evolutionarily constrained genes^1^. This recent study—and most other large-scale human genetics studies—was mainly composed of individuals of European (EUR) ancestry, and the generalizability of the findings in non-EUR populations remains unclear. To address this gap, we designed a custom sequencing panel of 161 genes selected based on the current knowledge of SCZ genetics and sequenced a new cohort of 11,580 SCZ cases and 10,555 controls of diverse ancestries. Replicating earlier work, we found that cases carried a significantly higher burden of rare protein-truncating variants (PTVs) among evolutionarily constrained genes (odds ratio = 1.48; *P* = 5.4 × 10^−6^). In meta-analyses with existing datasets totaling up to 35,828 cases and 107,877 controls, this excess burden was largely consistent across five ancestral populations. Two genes (*SRRM2* and *AKAP11*) were newly implicated as SCZ risk genes, and one gene (*PCLO*) was identified as shared by individuals with SCZ and those with autism. Overall, our results lend robust support to the rare allelic spectrum of the genetic architecture of SCZ being conserved across diverse human populations.

## Abstract (web summary)

Targeted sequencing finds a higher burden of rare protein-truncating variants in constrained genes among schizophrenia cases of diverse ancestries. Meta-analyses with existing datasets show that this excess burden is consistent across five ancestral populations.

## Main

SCZ is a severe, chronic psychiatric illness associated with lifelong progression and early mortality^2–4^. The genetic architecture of SCZ includes clear contributions from common single-nucleotide polymorphisms (SNPs)^5^, large copy number variants (CNVs)^6^ and rare PTVs^1,7–14^. Among these, rare PTVs provide unique value by linking disease risk to individual genes unambiguously. Most recently, the Schizophrenia Exome Sequencing Meta-Analysis (SCHEMA) Consortium increased the sequenced sample size for rare PTV investigations to 24,248 SCZ cases and 97,322 controls, established the rare PTV enrichment in genes under strong evolutionary constraint and identified ten genes with excess burden of rare PTVs in cases compared with controls^1^. When considered alongside earlier studies, these results suggest that, with greater sample sizes, additional SCZ genes harboring rare PTVs will be discovered. Whole-exome sequencing (WES) and whole-genome sequencing (WGS) remain cost prohibitive when applied at large scales, and targeted sequencing of carefully chosen genes is an alternative approach to rapidly achieve the required sample size for novel risk gene discovery.

Most large-scale human genetics research initiatives to date have failed to include diverse populations. Over 80% of genome-wide association study (GWAS) participants are of EUR ancestry, despite this group comprising less than one-quarter of the total human population^15,16^. Studies of mental illness have contributed to this disparity with almost exclusively EUR GWAS cohorts despite the roughly equal prevalence of psychiatric disorders worldwide^17^. The limited evidence from SCZ GWASs and CNV studies of non-EUR populations suggests broadly shared genetic architecture with that of EUR populations, but ancestry-specific effects, such as the major histocompatibility complex locus in EUR populations, are also present^18–24^. For rare genetic variants, findings on a broad range of complex human traits have been largely consistent across populations^25–30^. Evidence for ancestry-specific rare variant effects is limited but starting to emerge, such as *TMEM136* and serum lipid measurements in individuals of South Asian (SAS) ancestry^25^. No studies have yet shown the effect of rare PTVs in diverse ancestries for SCZ.

**Fig. 1:**
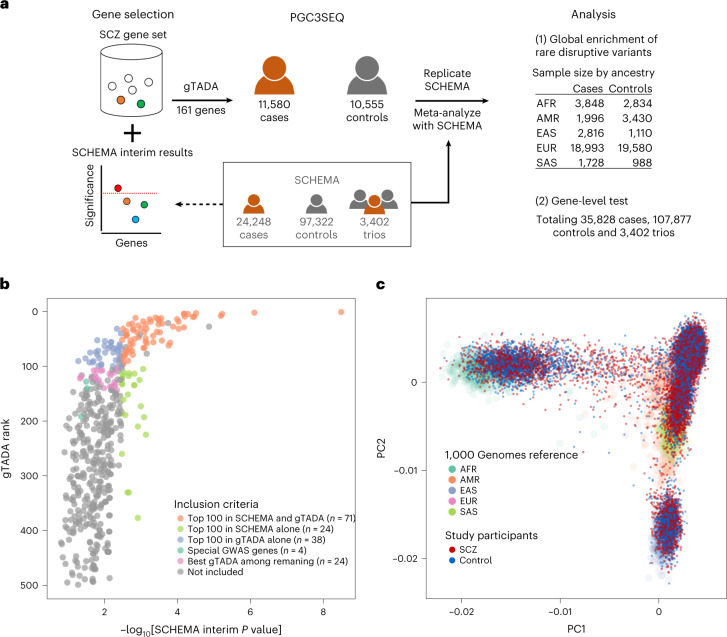
Study design and cohort ancestry composition. **a**, Overview of the study design. **b**, Gene selection for the targeted sequencing panel. Genes were selected based on a combination of previous association statistics (SCHEMA), gTADA rankings and GWAS associations. Specially, we included: (1) genes in the top 100 based on the gTADA rank and/or the SCHEMA *P* value (top 100 in SCHEMA and gTADA, top 100 in SCHEMA alone and top 100 in gTADA alone; total *n* = 133 genes); (2) genes with evidence for association with SCZ in both GWASs and SCHEMA (special GWAS genes; *n* = 4 genes); and (3) an additional 24 genes that had the best 24 gTADA rankings of the remaining genes with a burden *P* value of <0.05, to fill up the target panel. The *x* axis shows the gene-level *P* value using SCHEMA interim data, based on which the panel was constructed (different from the final published version). The *y* axis shows the gTADA rank of genes. Only the top 500 genes are plotted for a clear display. Some highly ranked genes were excluded (gray dots) due to logistic issues during panel construction. **c**, PGC3SEQ ancestry composition. PGC3SEQ samples include substantial non-EUR ancestry. The first two principal components (PCs) are plotted along each axis, colored by SCZ case versus control status. 1000 Genomes samples are colored by super-population.

Here, to diversify populations in SCZ studies and achieve sufficient power to discover novel risk genes, we designed a custom sequencing panel of 161 putative SCZ genes and applied it to case–control cohorts totaling 22,135 individuals from diverse ancestries (40% non-EUR; Fig. 1 and Supplementary Table 1). This study, outlined in Fig. 1a and hereafter referred to as the Psychiatric Genomics Consortium Phase 3 Targeted Sequencing of Schizophrenia Study (PGC3SEQ), was limited to cohorts that were not part of earlier SCZ sequencing initiatives such as SCHEMA. In constructing the sequencing panel, we used a data-driven algorithm to synthesize current knowledge of the genetic architecture of SCZ, including a preliminary version of the SCHEMA gene-level burden statistics^31,32^, with the goal of enriching for genes likely to harbor excess rare PTVs in SCZ that had not reached exome-wide significance due to a lack of power. This algorithm^33,34^ is a Bayesian framework that prioritizes genes by integrating gene-level burden statistics with gene membership in gene sets that have been implicated in SCZ (Fig. 1b and Supplementary Tables 2 and 3). The exonic regions of the 161 prioritized genes were sequenced on the Ion Torrent platform followed by rigorous quality control (Supplementary Figs. 1–6). Analyses comparing individuals with SCZ and controls were performed for rare PTVs (stop–gain, frameshift indels or essential splicing donor/acceptor) and deleterious missense variants (placed into tiers based on the missense badness, PolyPhen-2 and constraint (MPC) score^35^ (tier 1: MPC > 3; tier 2: MPC 2–3; nondamaging: MPC < 2), and synonymous variants were analyzed as a negative control. In our primary analysis, rare was defined as a minor allele count of ≤5 among the entire cohort. To maximize power, PGC3SEQ was further meta-analyzed with SCHEMA data (Supplementary Table 4 and Supplementary Fig. 7) and sequencing datasets for bipolar disorder and autism. We performed two broad types of analysis: (1) a global enrichment of all constrained genes on the custom panel (*n* = 80 genes) to investigate the overall role of rare disruptive variants in diverse ancestries; and (2) gene-level burden tests to identify novel SCZ risk genes.

**Fig. 2:**
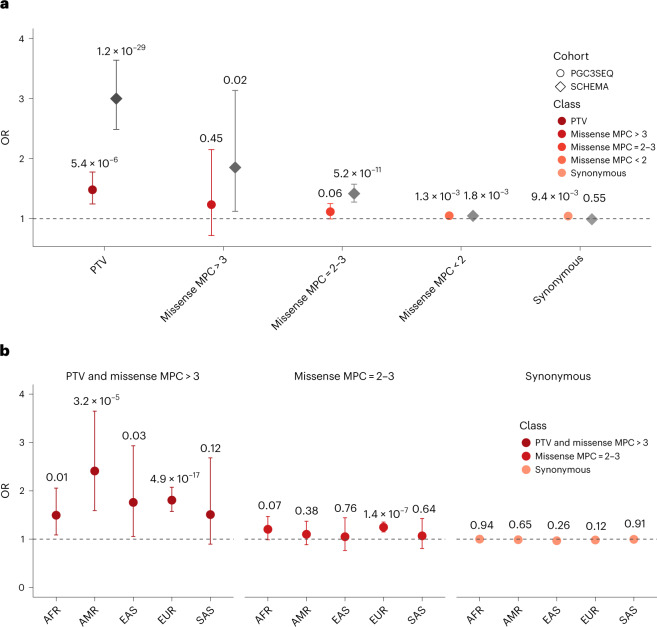
Global enrichment in 80 panel genes under strong constraint (pLI > 0.9). **a**, Case–control enrichment of rare (minor allele count ≤ 5) protein-truncating, missense and synonymous variants in all ancestries combined. The PGC3SEQ results were derived from 11,580 individuals with SCZ and 10,555 controls and are shown in red/orange. We conducted the same analysis in the SCHEMA samples (shown in gray; 19,108 cases and 18,001 controls) that we had access to for comparison. **b**, Ancestry-stratified rare variant (MAF < 0.1%) enrichment in the meta-analysis of PGC3SEQ and SCHEMA (29,381 cases and 27,942 controls). Three groups of variants were analyzed: PTV + MPC > 3 missense variants (combined to increase the power); MPC = 2–3 missense variants; and synonymous variants. The data are presented as point estimates of enrichment ORs (dots) and 95% confidence intervals (bars). Two-sided *P* values were calculated using Firth logistic regression, controlling for five ancestry principal components and either the rare synonymous variant count (for PTV and missense variants) or the rare nonsynonymous variant count (for synonymous variants), to control for potential unknown technical biases.

PGC3SEQ SCZ cases carried a significantly higher burden of rare PTVs among the 80 constrained genes after adjusting for rare synonymous variant counts and five ancestry principal components (odds ratio (OR) = 1.48; *P* = 5.4 × 10^−6^; Fig. 2a and Supplementary Table 5), indicating an independent replication of the excess burden of rare PTVs observed in 3,063 constrained genes in SCHEMA. The higher effect size seen in PGC3SEQ compared with SCHEMA (OR_PGC3SEQ_ = 1.48 in 80 genes; OR_SCHEMA_ = 1.22 in 3,063 genes) demonstrates the effectiveness of the gene prioritization strategy used for PGC3SEQ. For the 80 genes available in both studies, the signal in PGC3SEQ was much attenuated compared with in SCHEMA (OR_PGC3SEQ_ = 1.48 versus OR_SCHEMA_ = 3.0; Fig. 2a), indicating that effect sizes are probably overestimated in SCHEMA. In contrast, tier 1 and 2 missense variants were not significantly enriched in cases relative to controls in PGC3SEQ. The effects of missense variants were directionally consistent with those in SCHEMA, indicating that the insignificant results may be due to a lack of power. The burden of rare synonymous variants, which were analyzed as a negative control, was significantly higher in those with SCZ relative to controls in PGC3SEQ but not in SCHEMA. Sensitivity analysis showed that this signal was due to an overall higher burden of rare coding variants in people with SCZ relative to controls in PGC3SEQ, rather than due to technical bias or variability between contributing cohorts (Supplementary Note and Supplementary Fig. 8). The global PTV enrichment in PGC3SEQ remained significant after accounting for this overall higher baseline burden (OR = 1.4; *P* = 1.2 × 10^−4^; Supplementary Fig. 8c and Supplementary Table 5).

We performed meta-analyses of PGC3SEQ and SCHEMA to test whether the global enrichment signal was consistent across diverse ancestries (*n* = 57,323; ancestry breakdown in Fig. 1a). We assigned samples to five ancestral super-populations, as defined in the 1000 Genomes Project (Methods). At the aggregate level, four of the five populations displayed a higher burden of rare disruptive variants (PTV + MPC > 3 missense) in SCZ cases compared with controls (*P* < 0.05; Fig. 2b (left) and Supplementary Table 6). Although we did not find a nominally significant enrichment in the fifth ancestral population (SAS), the magnitude of enrichment was similar to that in the African (AFR) population (OR = 1.5), indicating that nonsignificance is probably a power issue (Supplementary Note and Supplementary Fig. 9). When considered separately, PGC3SEQ and SCHEMA provided independent support for the ancestry-stratified enrichments (all ancestries had OR > 1 in both datasets; Supplementary Table 6). Indeed, the PGC3SEQ data alone showed nominal significance for admixed American (AMR), East Asian (EAS) and EUR populations, exempt from any potential effect overestimation in SCHEMA. Differences, if any, in the strength of enrichment between pairs of ancestral populations were not sizable enough to be detected as significant. Across the five ancestral populations, the burden of tier 2 missense variants was evaluated, although not significant in most (OR = 1.1–1.2; Fig. 2b, middle), whereas synonymous variants were not enriched in any (Fig. 2b, right).

Having replicated the global rare PTV enrichment in PGC3SEQ and established its conservation across diverse populations, we then tested individual genes for harboring an excess burden of rare PTVs in cases relative to controls. In the PGC3SEQ data alone, none of the 161 genes sequenced were significant after Bonferroni correction (0.05/161 = 3.1 × 10^−4^; Supplementary Tables 7–9 and Supplementary Fig. 10). The direction of effects of all genes was overall consistent with the directions observed in SCHEMA (binomial test, *P* = 0.016) and this observation became more pronounced when considering only those 44 genes with a SCHEMA *P* value of <0.01 (binomial test, *P* = 0.002). Of the ten significant genes identified in SCHEMA, nine were included in the PGC3SEQ panel (*GRIA3* was the lone exception). PGC3SEQ had enrichment of rare PTVs for these nine genes collectively (OR = 1.66; *P* = 0.03; 49 PTVs in cases versus 24 in controls) and two of the nine genes had *P* < 0.05 when considered individually (*RB1CC1* and *CUL1*; Table 1). Notably, *SETD1A*—the gene with the strongest signal in SCHEMA—had a nonsignificant, weakened enrichment in PGC3SEQ, suggesting an overestimation of its effect magnitude in SCHEMA (OR_PGC3SEQ_ = 1.6 versus OR_SCHEMA_ = 20.1). Another gene implicated by SCHEMA that did not find support in PGC3SEQ was *CACNA1G*. Among the nine SCHEMA genes on the PGC3SEQ panel, *CACNA1G* had the largest number of PTVs in PGC3SEQ (*n* = 19) yet an OR of 0.42, directionally inconsistent with its effect in SCHEMA (OR_SCHEMA_ = 3.1). Despite some evidence of winner’s curse, altogether the gene-level replication tests in PGC3SEQ suggest that many of the SCHEMA genes probably confer genuine SCZ risk, including those not yet reaching exome-wide significance.

**Table 1:** Attempted replication of the nine significant SCHEMA genes in PGC3SEQ

Gene	PGC3SEQ						SCHEMA	
	Number of PTV alleles in cases	Number of PTV alleles in controls	Number of alleles in cases	Number of alleles in controls	OR (PTVs)	Fisher’s exact test *P*	OR (PTVs)	*P*
*SETD1A*	9	5	23,160	21,110	1.64	0.431	20.1	2.00 × 10^−12^
*CUL1*	6	0	23,160	21,110	Infinity	**0.032**	36.1	2.01 × 10^−9^
*XPO7*	5	0	23,158	21,110	Infinity	0.064	52.2	7.18 × 10^−9^
*TRIO*	3	3	23,160	21,110	0.91	1.000	5.0	6.35 × 10^−8^
*CACNA1G*	6	13	23,160	21,110	0.42	0.105	3.1	4.57 × 10^−7^
*SP4*	1	0	23,150	21,104	Infinity	1.000	9.4	5.08 × 10^−7^
*GRIN2A*	0	1	23,152	21,104	0.00	0.477	18.1	7.37 × 10^−7^
*HERC1*	9	2	23,160	21,110	4.10	0.069	3.5	1.26 × 10^−6^
*RB1CC1*	10	0	23,148	21,108	Infinity	**0.002**	10.0	2.00 × 10^−6^
Total	49	24			1.66	0.027		

All *P* values are two sided.

Combining PGC3SEQ and SCHEMA (totaling 35,828 cases and 107,877 controls) via a *P* value-based meta-analysis of gene-level statistics, we identified two new genes at the exome-wide significance threshold (Table 2 and Supplementary Table 7): *SRRM2* (*P* = 7.2 × 10^−7^) and *AKAP11* (*P* = 4.2 × 10^−7^). In previous work, *SRRM2* has been shown to play a role in the tauopathy of Alzheimer’s disease^36–38^, and de novo mutations in this gene have been linked to developmental disorders^39^, while *AKAP11* was suggested as a *trans*-gene linking to a SCZ GWAS locus in a recent study^40^, which, together with our results, adds to examples of the convergence of common and rare variant associations in the same gene. A recent meta-analysis of SCHEMA and a bipolar disorder dataset also found exome-wide significance for *AKAP11* (ref. ^41^), suggesting a role for this gene in the shared etiology of SCZ and bipolar disorder. The current study consolidates the role of *AKAP11* in SCZ, independent of other psychiatric disorders.

**Table 2:** Novel exome-wide significant SCZ genes

Gene	Location	pLI^a^	PGC3SEQ			SCHEMA^b^		Meta *P*^c^	
			Number of PTVs	OR (PTVs)	*P*	OR (PTVs)	*P*	SCZ	SCZ and ASD
*AKAP11*	Chr13:42846289–42897396	0.98	17	4.26	0.014	5.25	8.28 × 10^−6^	4.15 × 10^−7^	–
*SRRM2*	Chr16:2802330–2822539	1	10	9.12	0.013	7.14	7.19 × 10^−7^	7.19 × 10^−7^	–
*PCLO*	Chr7:82383329–82792246	1	8	5.01	0.024	4.02	9.36 × 10^−4^	1.06 × 10^−5^	5.84 × 10^−8^

^a^Probability of loss-of-function intolerance.

^b^The SCHEMA *P* values were retrieved from SCHEMA summary statistics and represent the strength of evidence from both case–control and patient–proband trio (de novo mutation) data.

SCZ, meta-analysis of PGC3SEQ and SCHEMA; SCZ and ASD, SCZ further meta-analyzed with Autism Sequencing Consortium WES.

^c^Meta-analysis *P* values were determined by Stouffer’s method and weighted by sample size.

All *P* values are two sided.

Lastly, we meta-analyzed gene-level rare disruptive variant statistics from SCZ, autism spectrum disorder (ASD)^42^ and bipolar disorder^41^ to identify pleiotropic risk genes that are not detectable at the sample sizes attained by studies of any single disorder. This identified *PCLO* as a shared risk gene for SCZ and ASD (*P* = 5.8 × 10^−8^; Table 2). The result suggests that *PCLO* may be driving the common variant association at nearby loci reported in GWASs of SCZ^43^ and other psychiatric disorders^44–47^.

The major contribution that the PGC3SEQ study makes to the field of human genetics is demonstrating the cross-ancestry conservation of the risk conferred by a major class of genetic variation for the most severe adult mental illness. To date, the paucity of exome sequencing studies of non-EUR populations has impeded the field in developing a complete view of the genetic architecture of complex diseases, and has made it difficult to assess the degree to which rare PTV associations are susceptible to the well-known confounding effects of ancestry in GWASs and polygenic prediction studies^48–52^. Here we addressed this knowledge gap with respect to severe mental illnesses. In doing so, findings previously established in predominantly EUR cohorts have been extended to non-EUR populations for one of the major classes of genetic risk variation. This observation was not a foregone conclusion, especially since the targeted gene list was derived from SCHEMA—a study of predominantly EUR cohorts. In effect, PGC3SEQ showed that the burden signal in genes with the strongest evidence in EUR populations is conserved across non-EUR populations. Our findings are also timely information following the publication of SCHEMA, showing that some of the top genes implicated in that study are probably false positives.

There are limitations to the current study. The Ion Torrent technology is known to have decreased accuracy for indels involving homopolymer repeats of the same nucleotide^53^. We assessed the impact of such indels on our findings via a sensitivity analysis and found that excluding them would not change our conclusions (Supplementary Tables 10 and 11 and Supplementary Fig. 11). We used an interim version of the SCHEMA results for PGC3SEQ panel design, and this version is different from the published results due to changes in SCHEMA analytical strategy. Specifically, the interim SCHEMA statistics^31,32^ did not include de novo mutations from trios, used a different strategy to combine PTV and missense variants and were compiled before the incorporation of Genome Aggregation Database (gnomAD) controls. Comparing the interim and published SCHEMA results, gene ranks underwent nontrivial changes, with only 27 overlapping genes between the top 100 lists in the two versions. Consequently, our panel probably included more random noise than it would have if panel construction had waited until SCHEMA was complete. As WES studies of other diseases approach the sample size achieved for SCZ, and strategies are considered for how to increase power, the current report offers valuable lessons, and we note that results on datasets as large as 24,000 cases and 50,000 controls can still change substantially as more samples are added. The possibility of such changes makes the targeted panel approach vulnerable, and perhaps WES and WGS are the safest strategies despite their cost.

In summary, rare PTVs have a robust role in SCZ, and across ancestries their effect is consistently concentrated in genes under strong evolutionary constraint. The deconvolution of this overall contribution into individual genes that may have ancestry-specific effects will require the sequencing of more individuals of diverse backgrounds. Achieving diversity in human genetic research must be a top priority to prevent health disparities from worsening as findings from genetic research begin to be translated into clinical practice.

## Methods

### Cohorts

A brief description of the individual contributing sample collection of PGC3SEQ is available in the Supplementary Note, along with the institutional review boards that approved the sample collections. To ensure compatibility with Psychiatric Genomics Consortium definitions, we define cases as those having a diagnosis of SCZ or a schizoaffective disorder. A total of 23,352 samples selected to be nonoverlapping with SCHEMA as well as other previous and ongoing sequencing efforts in the field were identified and sequenced (Supplementary Table 1). The PGC3SEQ study protocol was approved by the Icahn School of Medicine at Mount Sinai ethical review board (16-00101).

### Gene panel construction

We intended to build a panel of putative SCZ risk genes from within which the majority of new discoveries from additional WES and WGS would come. To this end, we applied both traditional burden statistics and the generalized/gene set transmission and de novo association test (gTADA) to the SCHEMA data.

#### Traditional burden statistics

For each gene in SCHEMA, the enrichment statistics of rare variants in cases compared with controls were calculated using Fisher’s exact test separately for PTVs and damaging missense variants, then the two classes of variants were combined using meta-analysis to generate a gene-level *P* value. Of note, this gene-level *P* value is different from that in the SCHEMA publication, which used a slightly different strategy in combining PTVs and missense variants, additionally incorporated evidence from de novo mutations using trio data and included external gnomAD controls. Such analysis strategy changes in the later stage of SCHEMA have led to nontrivial changes in gene rank, which may impact the power of our panel to implicate disease genes.

#### gTADA

gTADA is a generalized Bayesian framework where de novo and rare variant case/control data are integrated with gene-level external information to identify risk genes for neuropsychiatric disorders^33,34^. We first sought to identify gene sets associated with SCZ in SCHEMA. Through curation of the literature, we identified an initial set of ~160 candidate gene sets. Next, each set was tested independently for association with SCZ in SCHEMA data using gTADA. From all of the sets tested, we identified 27 significantly enriched gene sets. We then calculated a joint enrichment *Z* score from the marginal *Z* scores and the gene set correlation matrix and kept the 25 gene sets with positive joint *Z* scores (Supplementary Table 2). For each of the 25 sets retained, gene-level statistics (posterior probability of being a risk gene) were then calculated. The genes were then ranked by this metric and the mean ranking across the 25 ranks was calculated.

Combining traditional burden statistics and gTADA, genes in the top 100 based on the gTADA mean ranking across the 25 ranks or the top 100 based on the minimum ranking across the 25 ranks and/or the top 100 based on the burden test were included in the panel (Fig. 1b and Supplementary Table 3; *n* = 139 genes; six were later removed due to the logistics of designing the sequencing panel). We next included four genes with evidence for association with SCZ in both GWASs and SCHEMA, with the criteria being: gene burden test *P* value < 0.05; gene with a top 200 rank in gTADA; and gene start and stop positions spanning an SNP associated with SCZ in GWAS or, if not, gene located in a GWAS locus with fewer than or equal to ten genes. Finally, an additional 24 genes were chosen for inclusion by taking the best 24 gTADA rankings of the remaining genes with a burden *P* value < 0.05.

Based on the observation that gene-level rare single-nucleotide variant burden statistics have been consistent across ancestries in a wide range of diseases^18–24^, our targeted panel was expected to have broad utility across ancestries, even though its construction used EUR-dominant datasets. This was further consolidated by findings from our own ancestry-stratified analysis (Fig. 2b).

### Sequencing and variant calling

Ion AmpliSeq technology is an amplicon-based enrichment method for creating sequencing libraries. We used Ion AmpliSeq Designer version 6.13 to design amplicons that cover the exons of the 161 genes defined based on the Ion hg19 reference. The mean and median percentages of covered base pairs across all exons were 97.7 and 100%, respectively. Sequencing of the PGC3SEQ samples was performed on the Ion Torrent platform at Sema4 between June 2018 and April 2019. Sequencing plates were matched with respect to ancestry and case versus control composition whenever possible. The average sequencing depth across all samples was 224×. The Sema4 sequencing facility returned to the research team BAM files with flow signal and associated quality control metrics. Single-sample calling was performed using Torrent variantCaller version 5.8.0, which is specially optimized to exploit the underlying flow signal information generated by the Ion Torrent sequencing. Sites were left aligned and normalized and multiallelic sites were split into separate lines using BCFtools version 1.9 (http://samtools.github.io/bcftools/).

### Genotype-level quality control

We interrogated the call set with respect to a variety of quality control metrics and implement procedures to ensure rigorous quality control standards. In the absence of well-established quality control procedures specifically for Ion Torrent data, we drew on the idea of GATK’s variant quality score recalibration technique and developed a machine-learning genotype-level filter based on 177 quality metrics and annotation profiles, including Ion Torrent sequencing metrics such as QUAL, FMT/GQ and FMT/DP, allele-related metrics such as AF, HRUN and MLLD and coverage and allele frequency from the gnomAD database version 2 (https://gnomad.broadinstitute.org). Considering that the majority of SCHEMA data with which we meta-analyzed were generated on the Illumina platform, we calibrated our Ion Torrent targeted sequencing data using a subset of the control samples (*n* = 1,347) with available Illumina WES data. Specifically, we used XGBoost version 1.3 (ref. ^54^) in Python version 3.7.3 to train the classifier in 70% of the Ion Torrent–Illumina paired data using Illumina as the ground truth. In the remaining 30% test set, the classifier achieved an area under the curve of 0.95, an accuracy of 95.3% and a false discovery rate of 4.4% for SNPs and an accuracy of 99.0% and a false discovery rate of 6.4% for indels. Applying the trained classifier to the test dataset improved the concordance between Ion Torrent and Illumina calls from 83.1 to 95.7%. We also compared our machine-learning classifier with a set of conventional hard filters and confirmed that the classifier performs unanimously better in all metrics considered (sensitivity, specificity, accuracy and false discovery rate).

Applying the machine-learning filter to the entire dataset, 83.2% of the calls were retained, and among the passed variants, 96% were SNPs and 4% were indels. Five out of 919 detected multiallelics passed the filter and were split into multiple biallelic variants. The proportion of calls that passed the filter among samples used for model training and testing (*n* = 1,347) and the remaining samples were similar (83.9 versus 83.1%, respectively). Likewise, the pass rate among sites that were covered by both Illumina WES capture and our sequencing panel (33.8% of the calls fell into these regions) and sites only covered by our panel were comparable (85.8 versus 81.8%), indicating that the machine-learning model generalized well to new samples and new genomic regions

### Sample- and site-level quality control

To identify low-quality and outlier samples, we examined per-sample sequencing quality metrics, including the number of mapped reads, average read depth across the panel, on-target rate and uniformity rate. We also examined sample-level call set characteristics, including the call rate, inbreeding coefficient, transition-to-transversion ratio at heterozygote sites, heterozygous-to-homozygous call ratio, total number of variants, number of SNPs and indels and number of singletons. We visualized the distribution of the above quality control metrics (Supplementary Fig. 1) and identified 94 low-quality/outlier samples that met either one of the following criteria: MappedReads < 400,000; MeanDepth < 40; OnTarget < 80; Uniformity < 65; MissingCallRate > 0.3; Inbreeding_F > 0.6; Het_Hom_Ratio < 0.6; Total_SNPs < 400; and Total_Indels < 10. The number of low-quality or outlier samples was not significantly different between cases and controls (55 out of 12,045 cases were low quality or outliers and 38 out of 11,212 controls were low quality or outliers; chi-squared test, *P* = 0.1878). All of the quality control metrics distributed similarly between SCZ cases and controls (Supplementary Fig. 2).

When combining data from single-sample calls, a no call at a particular site in a particular sample was deemed as a homozygous reference genotype if the depth at that site in that sample was greater than ten and missing otherwise. Lastly, we applied the site-level filters to exclude variants with a missing rate of >10%.

### Sample relatedness

We used the population structure-adjusted relatedness estimation methods PC-AiR and PC-Relate to estimate pairwise relatedness between samples. In addition to the quality control steps performed per previous sections, we further performed linkage disequilibrium pruning on the dataset and removed indels before relatedness estimation. Considering that the conventional kinship coefficient ranges for varying degrees of relatedness may not be appropriate when the estimates are from targeted sequencing data covering only a small fraction of the genome, we derived empirical boundaries based on the clustering of sample pairs on an identity-by-descent kinship scatterplot (Supplementary Fig. 3). The unrelated and related pairs were clearly separated into two clusters with distinct patterns (unrelated pairs: lower oval-shaped cluster; related pairs: upper left). We identified 1,096 pairs of genetic relatives and retained one sample from each pair according to the following prioritization scheme: (1) the sample has fewer genetic relatives in the entire cohort; (2) patient with SCZ; (3) the sample has available genome-wide SNP data; (4) the sample has self-reported sex information; and (5) the sample has fewer missing genotypes for variants with a minor allele frequency (MAF) of <0.1%. These measures yielded a total of 22,135 unrelated individuals for downstream analysis.

### Control for population stratification

We calculated ancestry principal components for the 22,135 unrelated individuals in PLINK version 1.9 (ref. ^55^) using 1,392 linkage disequilibrium-pruned common SNPs (MAF > 1%) that passed all quality control steps. Cases and controls were broadly matched on population structures (Supplementary Fig. 5a,b). The first five principal components were used in later association analysis to control for population substructure, based on the observation that: (1) the first five principal components explained 75% of the cumulative variance in the genetic variation among study participants; and (2) the ability of principal components to separate ancestral genetic backgrounds dissipated after the first five principal components (Supplementary Fig. 5c).

### Ancestry assignment

The genetic ancestry assignment of the PGC3SEQ participants was done by calculating principal components jointly with 1000 Genomes phase 3 participants (*n* = 2,501), followed by a *K*-nearest-neighbor classification using the top three principal components. We restricted the analysis to 1,372 linkage disequilibrium-pruned common SNPs (MAF > 1%) that were present in both the study dataset and the reference dataset (1000 Genomes). The reference data were first cleaned and quality controlled using PLINK by filtering for missingness per individual (<10%) and missingness per SNP (<10%) and then subsetted to the variant set that passed all of the quality control filters in the PGC3SEQ cohort. The cleaned reference and study datasets were harmonized, combined and pruned for linkage disequilibrium, then input into PLINK for principal component analysis with default settings.

*K*-nearest-neighbor classification was used for ancestry assignment of the study participants. Cross-validation determined *K* = 5 and the first three principal components could best classify participants into five super-populations (AFR, AMR, EAS, EUR and SAS). Applying the trained classification model, we assigned each study participant to the super-population that included the most of the participant’s five neighbors. About half of our study participants had self-reported ancestry and ethnicity data, which were broadly consistent with their genetically inferred ancestry. There was reasonable concordance between the country of origin of the sample collection and assigned ancestries (Supplementary Fig. 6).

We then ran another round of principal component analysis for each global population separately to generate ancestry-specific principal components, identified ancestry-specific outliers on the principal component plots and removed the outliers and recalculated the principal components until no obvious outlier existed. After two rounds of recalculation, two EAS and seven SAS individuals were flagged as outliers within ancestry and were not included in the analysis in which stratification by population was performed.

### Variant annotation

We employed the same variation annotation workflow as was used in SCHEMA for ease of replication and comparison. Specially, annotation by LOFTEE (as implemented in the Variant Effect Predictor)^56^ was applied to variants that passed all quality control filters, and the analysis was restricted to the canonical transcript with the most damaging annotation. The three broad types of coding variants analyzed were: (1) PTVs, defined as any mutation that introduced a stop codon, changed the frame of the open reading frame or introduced a change at a predicted splice donor or splice acceptor site; (2) missense variants, which included any single-nucleotide variant that caused an amino acid change; and (3) synonymous variants, which resulted in no amino acid change, as a negative control. Missense variants were further partitioned into groups with increasing deleteriousness based on the MPC score annotation^35^. Tier 1 missense variants had an MPC score of >3, tier 2 missense variants had an MPC score of 2–3 and an MPC score of <2 indicated nondamaging missense variants. The use of MPC as the missense classifier was based on the SCHEMA results that were compared with Combined Annotation Dependent Depletion and PolyPhen; MPC most powerfully prioritized damaging missense de novo variants in ASD and developmental delay/intellectual disability trios^1^.

### Use of SCHEMA data

SCHEMA is a large multisite collaboration aggregating, generating and analyzing high-throughput exome sequencing data of individuals with SCZ and controls to advance gene discovery. We accessed the post-quality control data of a subset of SCHEMA case–control samples with appropriate sharing permissions at the time of this work and did not reperform genotype- and sample-level filtering. Specifically, the controls from gnomAD, as included in SCHEMA, were not used in the current study due to data sharing restrictions. After excluding 216 samples detected as genetic duplicates with a PGC3SEQ sample, the available SCHEMA datasets contained 19,108 cases and 18,001 controls (Supplementary Table 4). We used the genetic ancestry label for each individual determined by the SCHEMA analysis team and, within each ancestral group, calculated population-specific principal components using linkage disequilibrium-pruned SNPs with a MAF of >1%, a call rate of >95 and a Hardy–Weinberg *P* value of 1 × 10^−6^. Using a similar procedure to that used in the PGC3SEQ data analysis, we detected and removed 24 outlier samples from the EAS group. Supplementary Fig. 7 shows the ancestral composition of the SCHEMA cohort and Supplementary Table 4 displays the number of SCHEMA cases and controls used for this study by original sample collection.

### Statistical approaches for global enrichment across constrained genes

We defined rare variants as those with a minor allele count of ≤5 in the entire sample for any ancestry-combined analysis and lifted this threshold to MAF < 0.1% in ancestry-stratified analysis to preserve power. We counted the number of rare variants by annotation type observed in each participant in individual genes and added up the counts across the 80 constrained genes. The association between rare variant burden in the gene set of interest and SCZ status was tested using logistic regression with Firth’s penalized likelihood method to account for sparse data^57^, while adjusting for ancestry principal components and baseline rare variant burden. The first five global principal components were used in the ancestry-combined analysis and the first four principal components calculated within each ancestry were used in ancestry-stratified analysis. The baseline rare variant burden was used to control for technical and biological differences between cases and controls. To ensure a minimum correlation between the baseline burden and the burden of interest, we used the rare synonymous variant count as the baseline burden when the burden of interest was a PTV or missense variant and the rare nonsynonymous variant count as the baseline burden when the burden of interest was a synonymous variant. The significance threshold for the enrichment analysis was determined using the Bonferroni method, correcting for the five annotation classes tested (PTVs, the three missense groups and synonymous variants); that is, 0.05/5 = 0.01. *P* < 0.05 was used for nominal significance.

Using the available individual-level SCHEMA data, we performed global enrichment tests across the 80 constrained genes using similar approaches as in the PGC3SEQ analysis. Specifically, we used logistic regression with Firth’s correction and adjusted for ancestry principal components, sex, sequencing cohort and baseline rare variant burden. The first five global principal components were used in the ancestry-combined analysis and the first four principal components calculated within each ancestry were used in ancestry-stratified analysis.

Four of the global populations (AFR, AMR, EUR and EAS) had *n* > 100 in both PGC3SEQ and SCHEMA and we used inverse variance-weighted meta-analysis to combine their odds ratios in the two cohorts (sample size by population in Fig. 1a). To balance the power reduction due to sample stratification, we relaxed the definition of rare variants to include those with a MAF of <0.1% (compared with a minor allele count of ≤5 in the ancestry-combined analysis). In the full SCHEMA cohort, missense variants with MPC > 3 had a global signal on par with PTVs^1^; therefore, we grouped these two types of variants together in our analysis of both cohorts to further increase the power. Only PGC3SEQ contributed to the analysis of the SAS population.

### Statistical approaches for gene-based tests

Gene-based tests aggregate the effects of multiple rare variants and can increase the power to detect genetic associations^58^. It is reasonable to assume that rare disruptive variants in a gene all have the same effect direction (variant alleles associated with higher risk) and under this scenario a burden test is appropriate. Considering the sparsity of the observed count data, we used Fisher’s exact test to compare the burden of PTVs in cases and controls and computed two-sided *P* values. The total disruptive burden per gene was quantified by adding up all PTVs (or synonymous variants, as a negative control) annotated to the gene. Different from SCHEMA, we did not incorporate missense variants because they were not significantly enriched globally (Fig. 2a). We did not pursue a meta-analysis of the PTV and MPC > 3 variants because the extremely low number of MPC > 3 variants prohibited a reliable estimation of their effect magnitude, which would be used as weights in a meta-analysis. Although Fisher’s exact test is not able to accommodate covariates such as ancestry principal components and baseline burden, this did not adversely affect our analysis as the *Q*–*Q* plot showed no sign of inflation in the statistics (Supplementary Fig. 10, top row).

In the gene-level analysis of SCHEMA, case–control cohorts and trio cohorts were meta-analyzed, and rare variants found in both types of cohort were not double counted. We combined gene-level *P* values from PGC3SEQ and SCHEMA (summary statistics obtained from the SCHEMA publication) using signed Stouffer’s method, with the sign of the *Z* scores being the effect direction of the PTVs and the weights of each study calculated as:

$$\frac{4}{{\frac{1}{{\# {\rm{cases}}}} + \frac{1}{{\# {\rm{controls}}}}}} + (\# {\rm{trios}}\;{\rm{in}}\;{\rm{SCHEMA}})$$

The above equation applies equal weight to the case–control data and trio data. Since only a subset of genes had de novo mutations in SCHEMA trios and the number of trios was small relative to the case–control sample size, fine-tuning weights would not meaningfully change our results. This meta-analysis totaled 35,828 SCZ cases and 107,877 controls, representing the largest SCZ sequencing dataset to date. The exome-wide significance level was determined to be 0.05/(23,321 tests performed in SCHEMA + 161 tests performed in PGC3SEQ) = 2.13 × 10^−6^. As expected, the meta-analysis *P* values deviated substantially from the null (Supplementary Fig. 10, middle left), consistent with an enrichment of risk genes in the targeted panel. Gene-level synonymous variant *P* values displayed the expected null distribution (Supplementary Fig. 10 (middle right) and Supplementary Table 9), assuring that the gene-level PTV results were free from technical or methodological artifacts agnostic to variant annotation.

We then combined the two SCZ cohorts with the WES datasets of two other psychiatric diseases to identify genes shared across diagnoses. The two studies from which we obtained summary statistics were: (1) the latest release of the Autism Sequencing Consortium (ASC)^42^ (and we further converted the gene-level *q* values to *P* values); and (2) the WES of bipolar disorder by Palmer et al.^41^. Meta-analysis was performed similarly as above and the same exome-wide significance threshold was also applied (2.13 × 10^−6^). We noted some degree of control overlap between these studies (for example, SCHEMA and ASC both included Swedish controls from the same collection). As the overlap between SCHEMA and ASC consists only a small fraction of the entire sample, our analysis (and the discovery of *PCLO*) should only be minimally affected. The controls overlapping between SCZ and bipolar disorder are expected to be greater per contributing cohort makeup, although we did not identify any new genes.

### Reporting summary

Further information on research design is available in the Nature Portfolio Reporting Summary linked to this article.

## Online content

Any methods, additional references, Nature Portfolio reporting summaries, source data, extended data, supplementary information, acknowledgements, peer review information; details of author contributions and competing interests; and statements of data and code availability are available at 10.1038/s41588-023-01305-1.

## Supplementary information

Supplementary Information Supplementary Note, Figs, 1–11 and Tables 10 and 11.

Reporting Summary

Peer Review File

Supplementary Tables Supplementary Tables 1–9.

## Data Availability

We describe all of the datasets in the Methods and Supplementary Information. The raw PGC3SEQ genotype and phenotype datasets are permitted to be distributed at the individual level and we have deposited the data in the database of Genotypes and Phenotypes under accession number phs003138.v1.p1. We provide the aggregated variant counts at the gene and gene set level in Supplementary Tables 1–9. SCHEMA summary-level data are available online for viewing and download (https://schema.broadinstitute.org). SCHEMA individual-level whole-exome sequence data are hosted on the controlled-access Terra platform (https://app.terra.bio/) and shared with the collaborating study groups. Requests for access to the controlled datasets are managed by data custodians of the SCHEMA Consortium and the Broad Institute and are sent to sample contributing investigators for approval. The gnomAD database can be accessed at https://gnomad.broadinstitute.org.

## Code availability

The software and code used in this study are described in the Methods. In brief, we used Torrent variantCaller version 5.8.0 to call variants from the raw sequence data. For quality control and preprocessing, we used XGBoost version 1.3 in Python version 3.7.3, BCFtools version 1.9 and PLINK version 1.9. Reanalysis of the SCHEMA cohort was performed using Hail 0.1 and 0.2 (https://hail.is/). Main analyses of the PGC3SEQ data and their meta-analysis with SCHEMA were performed using R version 3.6 with various libraries. Visualization was generated with ggplot2 version 3.3.
