# Supplementary material for: Schizophrenia risk conferred by rare protein-truncating variants is conserved across diverse human populations
Source: Nat Genet. 2023 Mar 13;55(3):369–76. doi: 10.1038/s41588-023-01305-1 (PMC10011128; doi:10.1038/s41588-023-01305-1)
Supplement: Supplementary file 1 — Supplementary Note, Figs, 1–11 and Tables 10 and 11. [file 41588_2023_1305_MOESM1_ESM.pdf]

nature genetics

Letter

https://doi.org/10.1038/s41588-023-01305-1

Schizophrenia risk conferred by rare protein-truncating variants is conserved across diverse human populations

In the format provided by the authors and unedited

Supplementary Note

Table of Contents

Description of the analysis-ready dataset 1

Scrutinizing the global enrichment signal of synonymous variants 1

Power analysis of the global enrichment test by ancestry 2

Sensitivity analysis of Indels 2

Detailed cohort description 2

Reference 8

Supplementary Figures 9

Supplementary Tables 20

Description of the analysis-ready dataset

After all QC steps, the analysis-ready dataset comprised the genetic data of 22,135 unrelated individuals (11,580 cases, 10,555 controls) at 92,813 variants on 161 genes. We compared our call set with two Illumina sequencing datasets (SCHEMA and BioMe in Supplementary Figure 4), and observed that our dataset is well comparable in terms of SNP/Indels proportions, MAF distribution, and variant annotation type composition.

Scrutinizing the global enrichment signal of synonymous variants

By definition, synonymous variants and non-damaging missense variants (MPC<2) have no functional consequences on gene products, yet we observed a slightly elevated burden in SCZ cases for these two classes (Figure 2A) which could not be explained by additionally adjusting for sequencing batches or additional lower-rank ancestry PCs. Stratified analysis showed that this signal was not driven by specific sample collections (Supplementary Figure 8 top) or ancestry groups (Supplementary Figure 8 bottom left). When adjusting for the total number of rare coding variants as the baseline burden, the two variant classes were no longer enriched (Supplementary Figure 8 bottom right), indicating that their signals reflect an overall higher burden of rare coding variants in SCZ cases. This is corroborated by the observation that synonymous variants with a higher allele frequency than those in our main analysis were not enriched (MAF<0.1% synonymous variants had OR=1.00 and p-value=0.98). The PTV signal was robust to the adjustment of the overall rare coding variant burden. The results we reported in the main text for any non-synonymous variants were obtained after adjusting for synonymous variant count, and should be robust to technical or methodological artifacts that would equally affect variants of any annotation.

Power analysis of the global enrichment test by ancestry

To aid with interpreting our ancestry-stratified analysis results, we conducted Monte Carlo based estimation of statistical power for detecting a significant enrichment signal at different sample sizes, using the osDesign R package [1]. Supplementary Figure 9 showed that under a reasonable assumption of a true OR=2, we have adequate power in all population groups except for SAS (power=0.75 in SAS).

Sensitivity analysis of Indels

A known limitation of the Ion Torrent sequencing technology relative to other sequencing technologies (e.g., Illumina) is inaccuracy in detecting the length of homopolymer repeats of the same nucleotide, potentially causing false indel calls at such genomic loci[2]. Multiple steps were taken to rule out this known limitation of the Ion Torrent sequencing technology as a confounding factor in the analyses performed for the current study.

First, the characteristics of indels called were compared between Ion Torrent and Illumina data for the 1,347 PGC3SEQ samples that were sequenced on both the Ion Torrent and Illumina platforms. The filtered indel calls had a consistency of 93.6% across the two platforms. Supplementary Figure 4 also shows a comparable indel proportion between our dataset and two independent datasets sequenced using Illumina.

Second, we conducted a sensitivity analysis of the global enrichment and the gene-level burden test with Indels overlapping homopolymer stretches excluded. These homopolymer Indels constitute 12.9% of all originally tested rare PTVs. The results are summarized in Supplementary Table10, Supplementary Table11, and Supplementary Figure 11. The global enrichment of PTV in SCZ cases as compared to controls was not affected by the exclusion of homopolymer Indels. When all Indels were excluded (50% of all rare PTVs), the enrichment was stronger with an increased OR=1.81. In the gene-level burden test, Individual genes had largely consistent effects when homopolymer Indels were included and when they were excluded. Among the twelve genes implicated in SCHEMA and/or PGC3SEQ, six of them had homopolymer Indels and the most notable change in gene's effect was observed for *SETD1A*. After removing homopolymer indels, the counts of rare PTV in cases/controls have changed from 9/5 to 8/2, and the OR has increased from 1.6. to 3.6. Both effect estimates did not significantly differ from 1 and were lowered than the OR_{SCHEMA} = 20.1.

Detailed cohort description

US1|Carlos N. Pato, Michele T. Pato

The cohort recruited participants as part of the Genomic Psychiatry Cohort (GPC), a study based at Rutgers University that recruited controls and cases living and being treated in local

communities and healthcare delivery systems. Cases were interviewed using the DI-PAD, a semi-structured clinical interview administered by mental health professionals. Inclusion criteria for cases included meeting lifetime diagnostic criteria for SCZ or schizoaffective disorder in accordance with the OPCRIT algorithms for DSM-IV, ICD-10 or DSM-5 criteria. Individuals reporting no lifetime symptoms indicative of psychosis or mania and who had no first-degree relatives with these symptoms were included as control participants. Exclusion criteria included any premorbid organic mental disorders and premorbid history of significant drug or alcohol dependence by DSM-IV/5 that confounds the diagnosis of SCZ. DNA was extracted from whole blood. All participants gave written informed consent and the IRB of the participating institutions approved the protocol.

UK|Michael C O'Donovan, Michael J. Owen, James T.R. Walters
This cohort contains the CLOZUK3 cases who were taking the antipsychotic clozapine and had received a clinical diagnosis of treatment-resistant schizophrenia which in the UK means lack of response to at least two other antipsychotics at standard therapeutic doses for at least 6 weeks. Through collaboration with Leyden Delta, who supply and monitor clozapine in the UK, we acquired DNA from routine blood monitoring samples as previously reported (Pardinas et al, Nature Genetics, 2018). The UK Multicenter Research Ethics Committee approved the study.

US2|Panos Roussos
Samples contributed by US2 were derived from three cohorts as part of the CMC study; the Mount Sinai NIH Brain Bank and Tissue Repository, the University of Pennsylvania Brain Bank of Psychiatric illnesses and Alzheimer’s Disease Core Center, and the University of Pittsburgh NIH NeuroBioBank Brain and Tissue Repository. In all cohorts, ethical approval was obtained from all participating sites, and all subjects provided informed consent. Tissue for the collection was dissected at each brain bank and shipped to the Icahn School of Medicine at Mount Sinai (ISMMS) for nucleotide isolation and data generation in one facility to reduce site-specific sources of technical variation. Postmortem tissue from schizophrenia and bipolar disorder cases were included if they met the diagnostic criteria in DSM-IV for schizophrenia or schizoaffective disorder, or for bipolar disorder, as determined in consensus conferences after review of medical records, direct clinical assessments, and interviews of care providers. Cases that had a history Alzheimer’s disease, and/or Parkinson’s disease, or acute neurological insults (anoxia, strokes, and/or traumatic brain injury) immediately prior to death, or were on ventilators near the time of death, were excluded.

MSSM samples - Mount Sinai NIH Brain Bank and Tissue Repository: Brain specimens are obtained from the Pilgrim Psychiatric Center, collaborating nursing homes, Veteran Affairs Medical Centers and the Suffolk County Medical Examiner’s Office. Disease diagnoses are made based on DSM-IV criteria and are obtained through direct assessment of subjects using structured interviews and/or through psychological autopsy by extensive review of medical records and informant and caregiver interviews. Consent is obtained from next of kin. The brain bank procedures are approved by the ISMMS IRB and exempted from further IRB review due to the collection and distributions of postmortem specimens.

Penn samples - University of Pennsylvania Brain Bank of Psychiatric illnesses and Alzheimer’s Disease Core Center: Brain specimens are obtained from the Penn Alzheimer's Disease Core Center prospective collection. Disease diagnoses are made based on DSM-IV criteria and obtained through a clinical interview by psychiatrist and review of medical records. All procedures for Penn are approved by the Committee on Studies Involving Human Beings of the University of Pennsylvania, and the use of control postmortem tissues was considered exempted research in accordance with CFR 46.101 (b), item 65 of Federal regulations and University policy.

Pitt samples - The University of Pittsburgh NIH NeuroBioBank Brain and Tissue Repository: Brain specimens are obtained during routine autopsies conducted at the Allegheny County Office of the Medical Examiner (Pittsburgh) following the consent of the next of kin. An independent committee of experienced research clinicians makes consensus DSM-IV diagnoses for all subjects on the basis of medical records and structured diagnostic interviews conducted with the decedent's family members. All procedures for Pitt samples have been approved by the University of Pittsburgh's Committee for the Oversight of Research involving the Dead and Institutional Review Board for Biomedical Research.

US3| Kerry J. Ressler

Samples contributed by US3 were part of a larger investigation of genetic and environmental factors in a predominantly African American (AA) urban population of low socioeconomic status with the purpose of determining how those factors may modulate the response to stressful life events. Research participants were approached in the waiting rooms of primary care of a large, public hospital (Grady Memorial Hospital in Atlanta, Georgia) while either waiting for their medical appointments or while waiting with others who were scheduled for medical appointments. Screening interviews, including the participants' demographic information (e.g., self-identified race, sex, and age) and psychiatric history were completed on site. DNA was extracted from saliva at Mount Sinai. Written and verbal informed consent was obtained for all participants and all procedures in this study were approved by the institutional review boards of Emory University School of Medicine and Grady Memorial Hospital, Atlanta, Georgia.

Germany| Annette M. Hartmann, Dan Rujescu

German samples were collected by separate groups within the MooDS Consortium in Mannheim, Bonn, Munich and Jena. In Bonn/Mannheim, cases were ascertained as previously described. Controls were drawn from three population-based epidemiological studies: PopGen, the Cooperative Health Research in the Region of Augsburg (KORA) study, and the Heinz Nixdorf Recall (HNR) study. All participants gave written informed consent and the local ethics committees approved the human subjects protocols. Additional controls were randomly selected from a Munich-based community sample and screened for the presence of anxiety and affective disorders using the Structured Clinical Interview for DSM-IV. Only individuals negative for the above-mentioned disorders were included in the sample.

US4| Charney W. Alexander

Founded in September 2007, BioMe is a biobank that links genetic and EMR data for more than 30,000 individuals recruited primarily in ambulatory care settings in the Mount Sinai Health System (MSHS) in New York City. An ethnically diverse, control subset of the BioMe samples was included in the US4 cohort. The current study was approved by the Icahn School of Medicine at Mount Sinai Institutional Review Board (IRB; approval 07-0529). All study participants provided written informed consent.

US5| Katherine E. Burdick

US5 consists of adult outpatients with a diagnosis of schizophrenia or schizoaffective disorder who provided informed consent for a cross-sectional study. We collected detailed diagnostic, clinical, and cognitive measures alongside blood collection for DNA and other genetic analyses.

US6| Sophia Frangou

Samples contributed by US6 included subjects recruited through the Mount Sinai Conte Center. Ethical approval was obtained from this site, and all subjects provided informed consent.

US7|Joseph D. Buxbaum

Samples contributed by US7 were comprised of patients ascertained in Israel. The study was approved by ethics committees in Sheba Medical Center (Israel). All subjects provided informed consent.

US8|Todd Lencz, Anil K. Malhotra

Patients with schizophrenia-spectrum disorders (including schizophrenia, schizoaffective disorder, or schizophreniform disorder) were recruited from the inpatient and outpatient clinical services of The Zucker Hillside Hospital, a division of the Northwell Health System. After providing written informed consent, the Structured Clinical Interview for DSM-IV Axis I disorders (SCID, version 2.0) was administered by trained raters. Information obtained from the SCID was supplemented by a review of medical records and interviews with family informants when possible; all diagnostic information was compiled into a narrative case summary and presented to a consensus diagnostic committee, consisting of a minimum of three senior faculty. Healthy comparison subjects were recruited by use of local newspaper advertisements, flyers and community Internet resources, and underwent initial telephone screening to assess eligibility criteria. The nonpatient SCID (SCID-NP) was administered to subjects who met eligibility criteria, to rule out the presence of an Axis I psychiatric disorder; a urine toxicology screen for drug use and an assessment of the subject's family history of psychiatric disorders were also performed. Exclusion criteria included (current or past) schizophrenia spectrum disorder, as well as current Axis I psychiatric disorder, psychotropic drug treatment, or substance abuse. Any subject deemed unable to provide written informed consent was also excluded.

Pakistan| Muhammad Ayub

[wait to hear back from contributor]

US9| Dheeraj Malhotra, Enrico Domenici

Roche cases were collected by Roche as part of clinical collaborations with hospitals and outpatient centers for eight PhaseII and Phase III, multi-center, randomized, double-blind, parallel-group, placebo-controlled study to evaluate the efficacy and safety of RO4917838 in patients with sub-optimally controlled symptoms of schizophrenia treated with antipsychotics, patients with an acute exacerbation of schizophrenia and in patients with prominent negative and disorganized thought symptoms. Cases were diagnosed according to DSMIV criteria, with medical record review by a trained psychiatrist. Cases gave informed written informed consent, IRBs at each collecting site and Roche ethics committee approved the human subjects protocol.

Australia| Janice M. Fullerton, Vaughan Carr

Samples contributed by the ASGC were collected as part of the Australian Schizophrenia Research Bank. Study participants were recruited in four Australian States (New South Wales, Queensland, Western Australia and Victoria) through hospital inpatient units, community mental health services, outpatient clinics and rehabilitation services, non-government mental illness support organizations, and a national multi-media advertising campaign (Loughland et al, ANZJP 2010; PMID:21034186). Ethical approval was obtained from all participating sites, and all participants provided informed consent. A subset of the ASRB sample were employed in a prior paper by the Schizophrenia Working Group of the PGC (Ruderfer et al, Cell 2018; PMID:29906448) under the cohort label "scz_asrb_eur".

Fiji| Bryan Mowry

The indigenous Fijian schizophrenia cases and healthy controls included here were part of a wider study to recruit, diagnostically ascertain and collect DNA samples from indigenous Fijian and Fijian Indian participants. Written, informed consent was obtained through procedures approved

by the institutional ethics committees and the Fiji Ministry of Health. Cases were recruited from St Giles Hospital, Suva the epicenter for psychiatric services across the Fijian archipelago. Cases included both inpatients and outpatients, who were contacted via an outreach community psychiatric nursing service. Controls were recruited from non-psychiatric outpatient clinics who were screened for mental health issues. 123 indigenous Fijian schizophrenia cases and 26 controls were sequenced for this study.

France| Claudine Laurent-Levinson, Dominique Campion
The cohort included two independent French schizophrenia cohorts. Firstly, case participants were unrelated Caucasian in- or out-patients who were recruited for the study of hyperprolinemia in SCZ patients. Cases were interviewed using the PANSS and appropriate sections of the Schedule for Affective Disorders and Schizophrenia, by licensed psychiatrists. Final DSM-III-R diagnoses were assigned by a group of trained psychiatrists based on the interview and available clinical records. We recruited control participants mainly from staff members. The study had ethics approval granted by local IRB ethic committee of Rouen, which permit inclusion of the data in meta-analyses. Blood samples were taken for DNA extraction. A subset of the Rouen- Pitié cohort consisted of 24 cases recruited separately in a study of childhood-onset SCZ in the Paris region. Cases with age at onset between 7-17 years were interviewed using the French version of the DIGS 2.0 by licensed psychiatrists. Participants were included in the genotyping cohort if the final DSM-IV diagnosis was SCZ or schizoaffective disorder depressed type, and the patient and family agreed to provide a blood specimen for genetic studies. The study was approved by the relevant ethics committee.

Indonesia| Dieter B. Wildenauer, Sibylle G. Schwab
Samples were obtained through the PsychChip genotyping initiative, and comprised samples from patients with schizophrenia admitted consecutively to psychiatric hospitals in the greater Jakarta area, who were informed about the study and were asked to sign the Informed Consent document for participation in interviews, blood withdrawal and subsequent genetic studies. The study was approved by the institutional review board of the University of Indonesia and by local Ethics Committees. Clinical consensus diagnosis of schizophrenia was made by psychiatrists according to the DSM-IV criteria. Non-psychiatric controls were recruited from students and staff of the University of Indonesia, Jakarta, and from the participating hospitals. Details of the study were previously reported (Schwab et al, Association of rs1344706 in the ZNF804A gene with schizophrenia in a case/control sample from Indonesia. Schiz Res 147: 45-52 (2013)

Italy1| Massimo Gennarelli, Luisella Bocchio-Chiavetto
The cohort includes more than 600 schizophrenia patients and not-affected volunteers of Caucasian ancestry for at least two generations, living in the Lombardy region of Italy. The collection of DNA samples and its sharing with the Icahn School of Medicine were approved by the Institutional Ethical Board and authorized by donors under informed consent. Patients admitted to the Psychiatry Unit of Brescia IRCCS Centro S. Giovanni di Dio Fatebenefratelli were enrolled if they had a DSM-IV-TR diagnosis of schizophrenia. Control subjects were randomly recruited from different sources (hospital visitors, cultural and elderly associations, trade unions, etc) and were screened for DSM-IV Axis I disorders. Only volunteers without a history of substance abuse or dependence and without a personal or first-degree family history of psychiatric disorders were enrolled in the study. Subjects who obtained a score lower than 27/30 at the Mini Mental State Examination were excluded as well.

Italy2| Antonio Rampino, Alessandro Bertolino
Samples contributed by University of Bari (Bari-Italy) included a total of 1,540 Caucasian white individuals, 913 healthy individuals (487 females) and 627 patients with SCZ (191 females).

Ethical approval was obtained from the Local Ethic Committee and all subjects provided informed consent accordingly. Whole blood was collected from all individuals and DNA for subsequent whole genome genotyping was extracted from whole blood.

Spain1| Celso Arango, Javier González-Peñas
Samples contributed were derived various cohorts from CIBERSAM (Centro de Investigación Biomédica en Red en Salud Mental, Spain). In all cohorts, informed consent signed by each participating subject or legal guardian and approval from the corresponding Research Ethics Committee were obtained. All of the cohorts, except three of them, were genotyped for a new schizophrenia GWAS at PGC (wave 3).

Spain2| Margarita Rivera
Samples contributed by the University of Granada (Spain) were derived from two cohorts. Ethical approval was obtained from both studies, and all participants provided informed consent. Patients were recruited as part of the GENIMS and PISMA studies. GENIMS was a cross-sectional clinical study in which participating patients were consecutive attendees to psychiatric outpatient clinics (Muñoz-Negro J.E. et al, Schizophr Res. 2015; 169(1-3):248-254). All were in a sSupplementary Tabletage of their disorder and on antipsychotic medication. Patients were all diagnosed by fully trained psychiatrists using the Structured Clinical Interview for DSM-IV Axis I disorder (SCID-I). Trained raters reviewed these interviews along with available clinical records to determine a consensus lifetime DSM-IV diagnosis of schizophrenia. Additional assessments included sociodemographic and clinical variables such as sex, age, educational level, employment, marital status, and years after onset. To estimate each participant's premorbid intelligence quotient (IQ), a Spanish version of the Barona index was used. The Spanish version of the PANSS was used to measure psychopathology, since PANSS is the standard scale valid and reliable for this purpose. Global functioning was assessed using the GAF. Inclusion criteria were as follows: 1) meet DSM-IV diagnostic criteria for SZ; 2) be older than 18 years; and 3) agree to participate. Exclusion criteria were as follows: 1) mental retardation and 2) any type of dementia. Control participants were recruited from the PISMA study, which has been reported elsewhere (Cervilla et al., Rev Psiquiatr Salud Ment 2016; 9(4):185-194). This was a cross-sectional study targeting a large representative stratified sample of community-dwelling Andalusian adults between 18 and 75 years of age. All provinces in the Andalusian community were included. Participants were administered the MINI by trained psychologists, which generated both DSM-IV and ICD-10 diagnoses. A saliva sample was obtained from each participant.

US10| Vishwajit L. Nimgaonkar
The inclusion criteria for cases were either a diagnosis of schizophrenia or schizoaffective disorder or, at Baltimore, schizophreniform disorder, according to DSM-IV criteria. The cases in Baltimore were recruited from inpatient and day hospital programs of Sheppard Pratt and from affiliated psychiatric rehabilitation programs. The cases in Pittsburgh were recruited from Western Psychiatric Institute and Clinic, Pittsburgh and additional psychiatric treatment facilities in a 500-mile radius of Pittsburgh. Patients were evaluated using structured diagnostic instruments, alongside medical records and informant interviews, where available. The control group was recruited from posted announcements at local health care facilities and universities in the same geographic area and settings where the schizophrenia participants were recruited. All participants provided written informed consent and the study was approved by the Institutional Review Boards of Sheppard Pratt, the University of Pittsburgh School of Medicine, and the Johns Hopkins School of Medicine following established guidelines.

Reference

1. Haneuse, S., Saegusa, T. & Lumley, T. osDesign: An R Package for the Analysis, Evaluation, and Design of Two-Phase and Case-Control Studies. *Journal of Statistical Software* **43,** 1–29 (2011).
2. Bragg, L. M., Stone, G., Butler, M. K., Hugenholtz, P. & Tyson, G. W. Shining a light on dark sequencing: characterising errors in Ion Torrent PGM data. *PLoS Comput Biol* **9,** e1003031 (2013).

Supplementary Figures

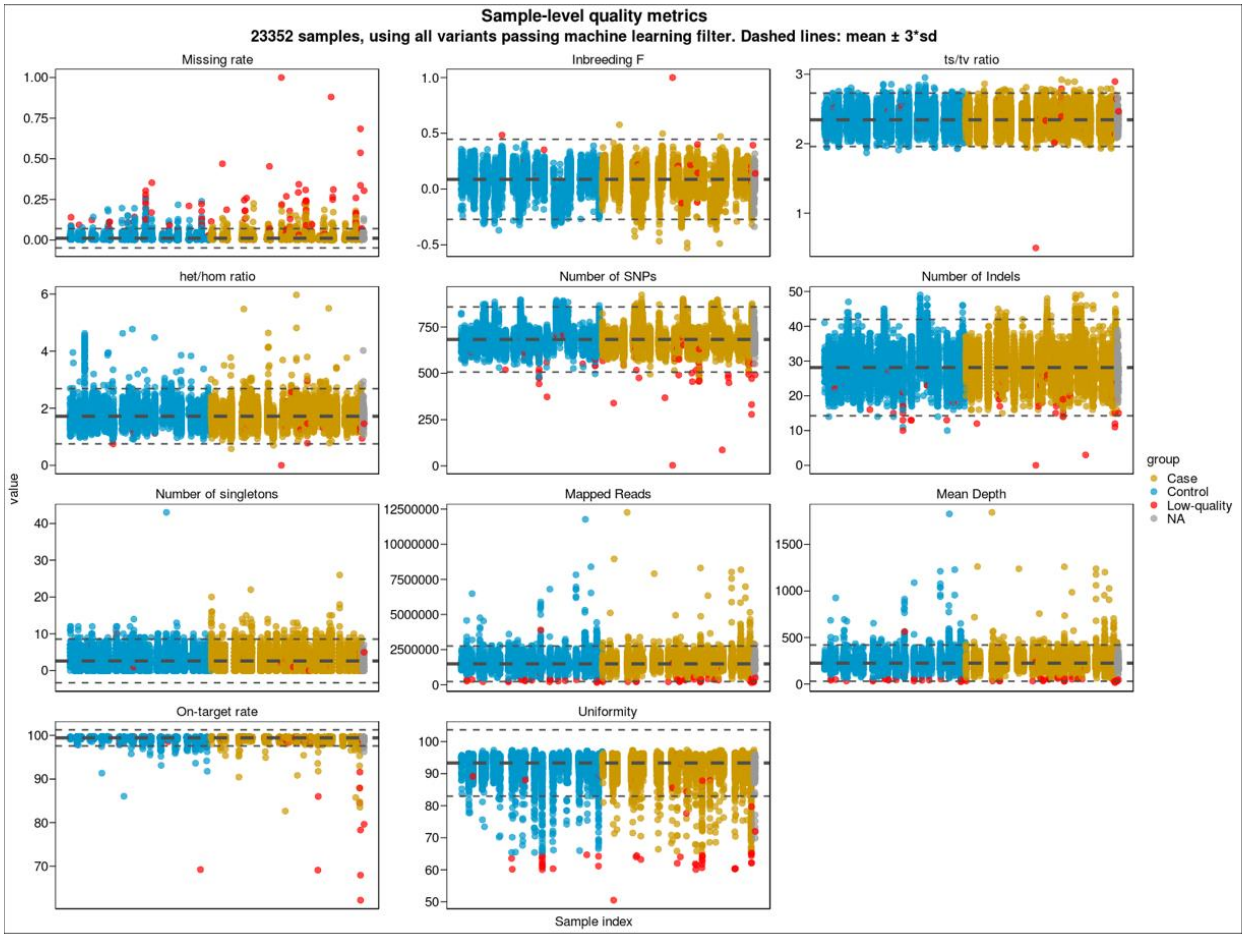

Supplementary Figure **1**. Sample-level sequencing and calling metrics with outlier and low-quality samples (N=94) are highlighted in red.

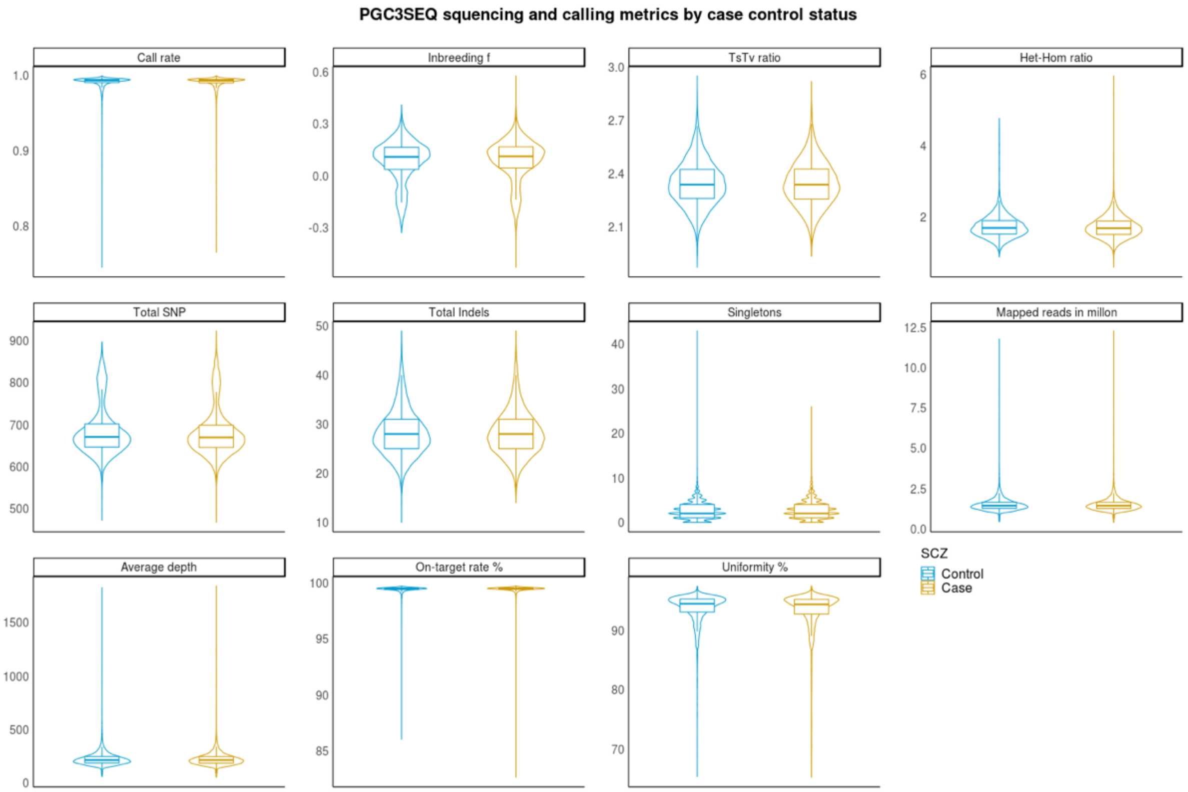

Supplementary Figure 2. Sample-level sequencing and calling metrics for 23,352 samples by case control status in the final PGC3SEQ sample set. In the box plot, the lower hinge is the 25% quantile, the middle line is the median, the upper hinge is the 75% quantile, the lower whisker extends to the smallest observation greater than or equal to the lower hinge − 1.5 × interquartile range (IQR) and the upper whisker extends to the largest observation less than or equal to the upper hinge + 1.5 × IQR.

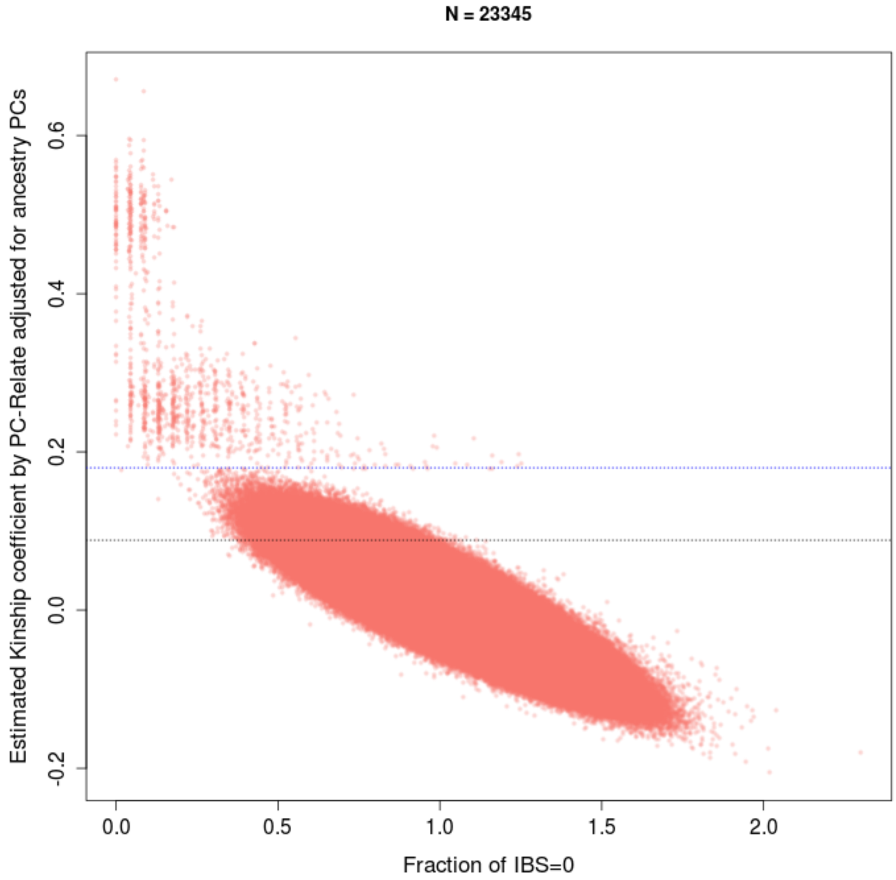

Supplementary Figure 3. Relatedness analysis of 23,352 PGC3SEQ samples using PC-relate. Y axis: kinship coefficients, x axis: the fraction of loci in which individuals share zero alleles IBS. The black (lower) dotted line marks the commonly used lower bound for second-degree relatives (kinship=0.0884). The blue (top) dotted line indicates the customized threshold used for determining unrelated pairs in PGC3SEQ.

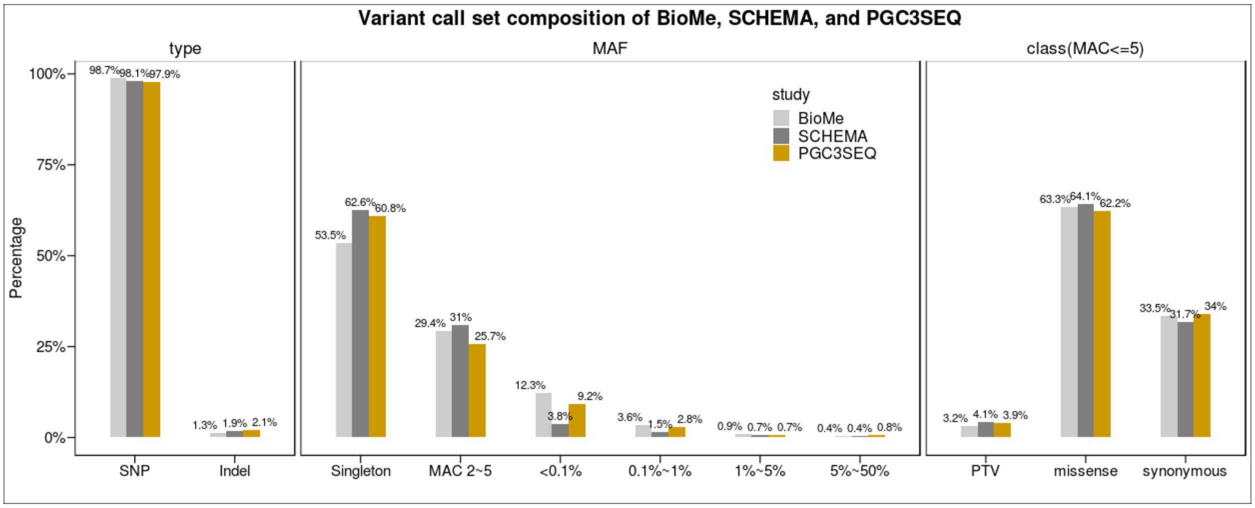

Supplementary Figure 4. Compare call set characteristics of PGC3SEQ with two sequencing datasets generated on the Illumina platform. BioMe consists the whole exome sequencing data of over 30,000 individuals recruited in a health care setting. For BioMe and SCHEMA, we first took the subset of the variants in the 161 genes that are also sequenced in PGC3SEQ. The proportion of SNP/Indel and different MAF bins are calculated in variants of any frequency and any annotation, while the functional annotation comparison is restricted to variants with MAC<=5.

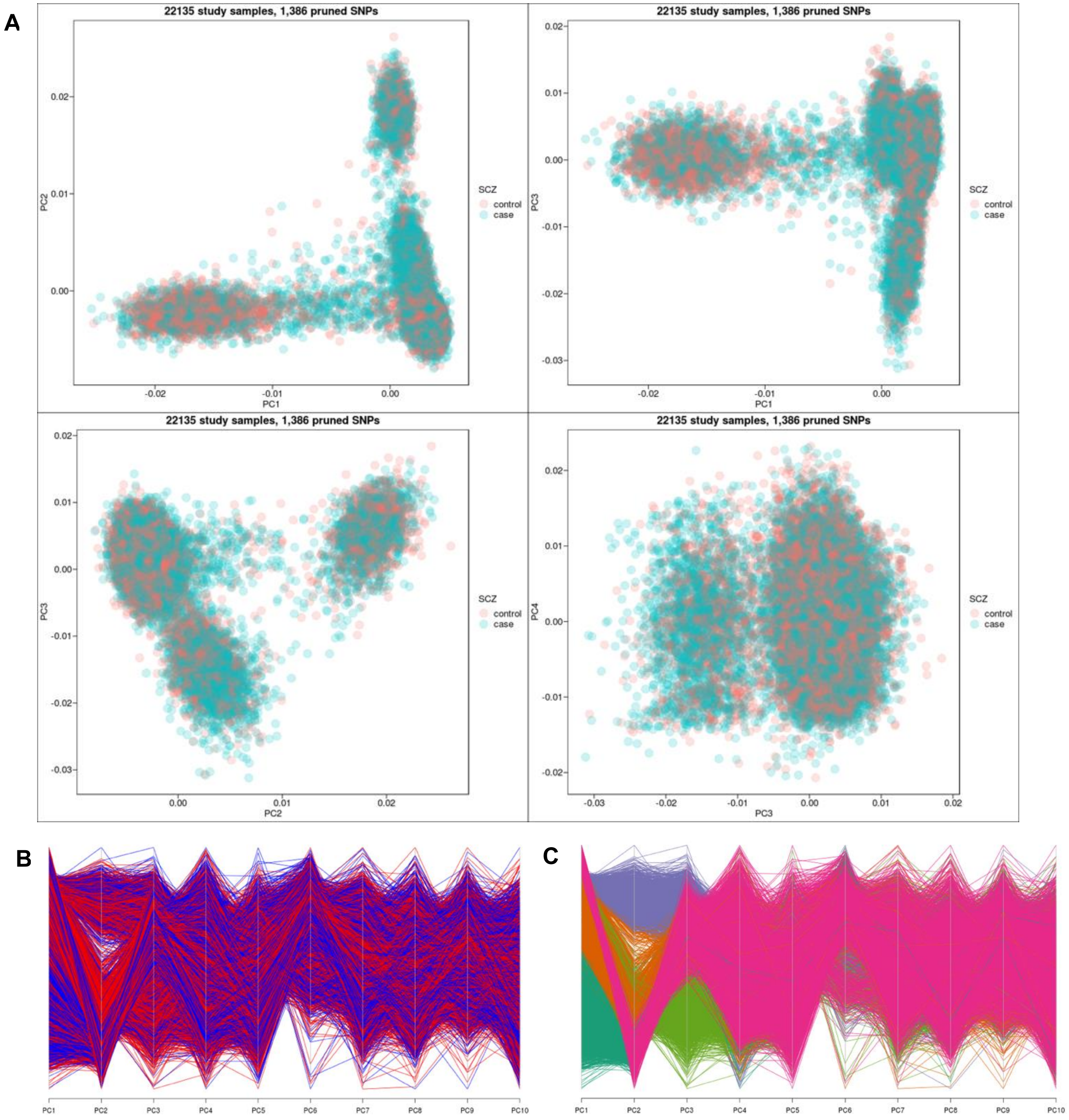

Supplementary Figure 5. (A) First four global PCs for SCZ cases (blue) and controls (red). (B) Parallel coordinate plot of PC1-PC10, colored by case control status. (C) Parallel coordinate plot of PC1-PC10, colored by genetically inferred ancestry.

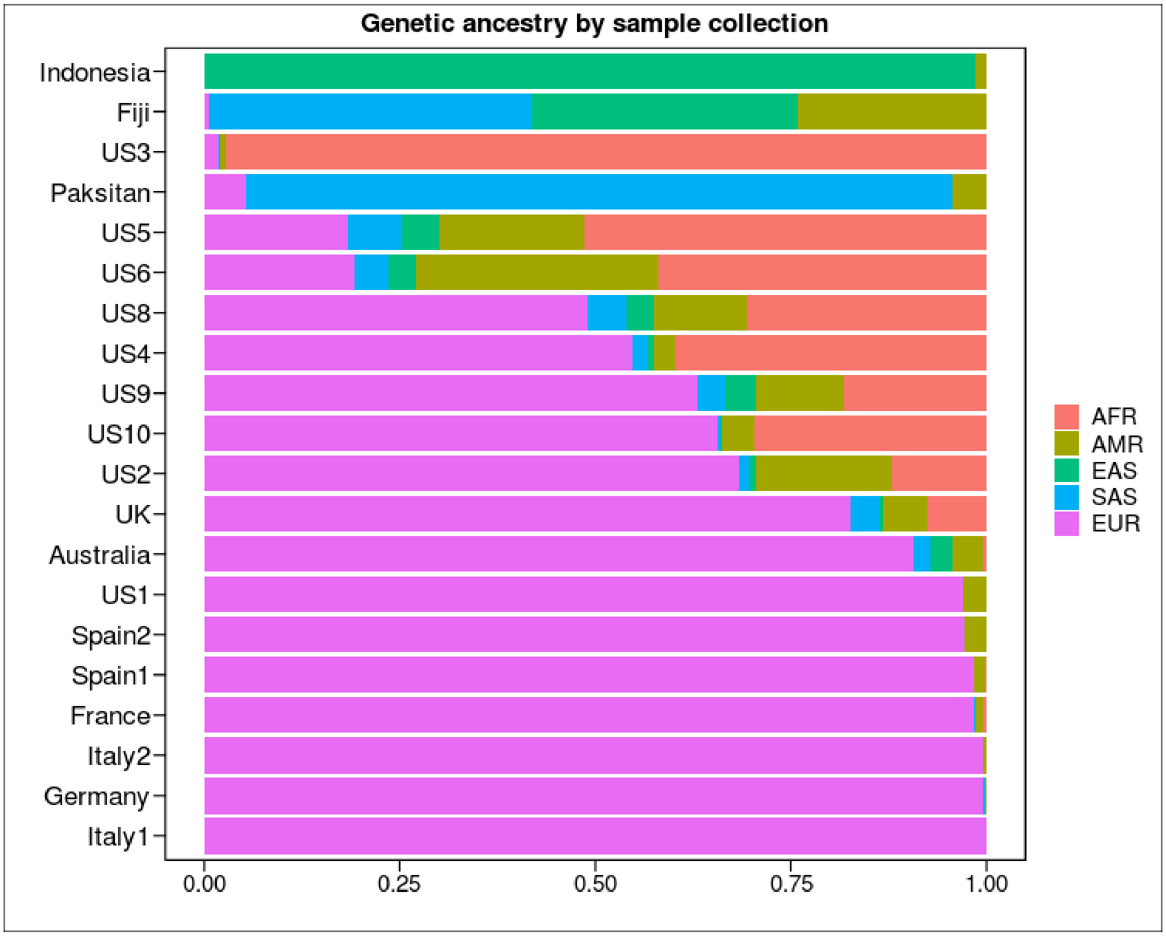

Supplementary Figure 6. Ancestry assignment by individual sample collection. Each sample is assigned a global ancestry by K-nearest neighbor classification using 1000 Genome subjects as a reference. There is reasonable concordance between country of origin and assigned ancestries.

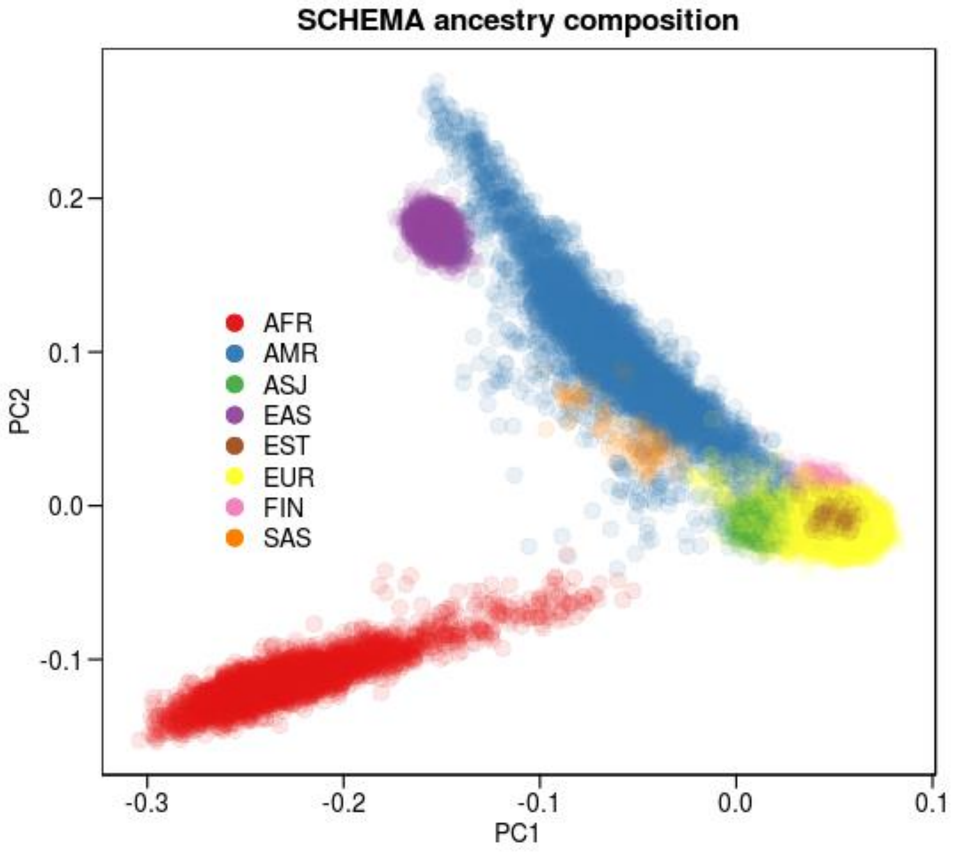

Supplementary Figure 7. Ancestry composition of the SCHEMA samples with available individual-level genetic data. The first two principal components are plotted along the axes, colored by genetically-inferred ancestry. AFR: African, AMR: Admixed American, EAS: East Asian, EUR: European, SAS: South Asian, ASJ: Ashkenazi Jewish, EST: Estonian, FIN: Finnish.

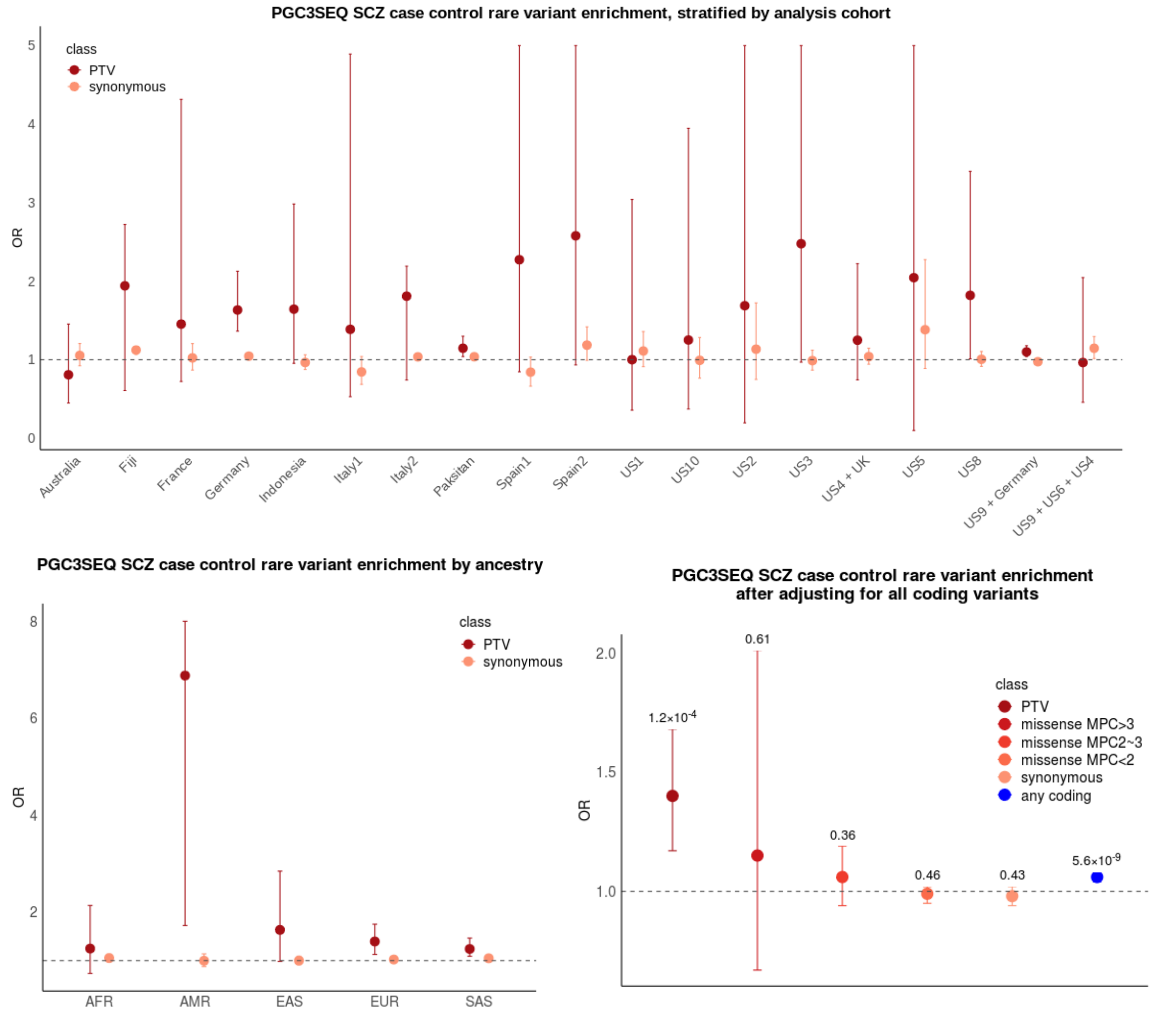

Supplementary Figure 8. Scrutinizing the enrichment signal of synonymous variants. Global enrichment in genes under strong constraint (pLI>0.9) stratified by **(top)** 19 analysis cohorts (some sample collections are combined to form case-control analysis cohorts) and **(bottom left)** five populations. Some sample collections are case-only or control-only and we recombined them to form 19 analysis cohorts. **(Bottom right)** Same as Figure 2A but instead adjusts for the overall burden of rare coding variants across the constrained genes (except for the class of any coding variant which is in blue). The blue dot shows that SCZ cases had a significantly higher overall burden of rare coding variants compared to controls, which gave rise to the enrichment signals of the synonymous and non-damaging missense variants in Figure 2A. After controlling for differences in this background burden, synonymous and non-damaging missense variants no longer showed a signal, while PTVs remained to be enriched at a similar magnitude. Data are presented as point estimate of enrichment OR (dots) and 95% confidence intervals (bars).

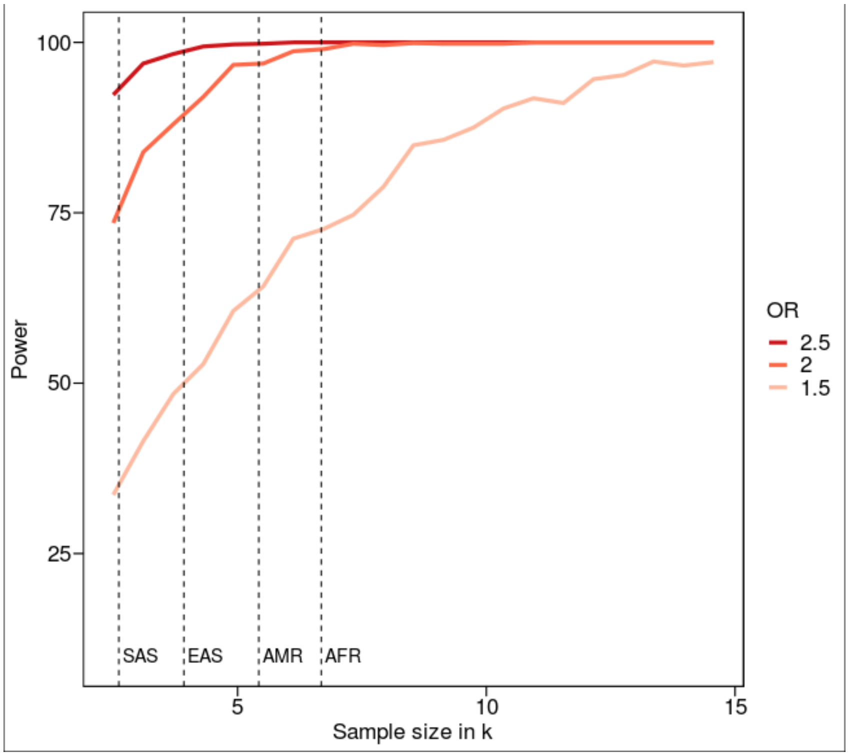

Supplementary Figure 9. Monte Carlo based power estimation of detecting a significant enrichment signal, given three different true ORs. Dotted lines indicate the current sample size for four non-European populations.

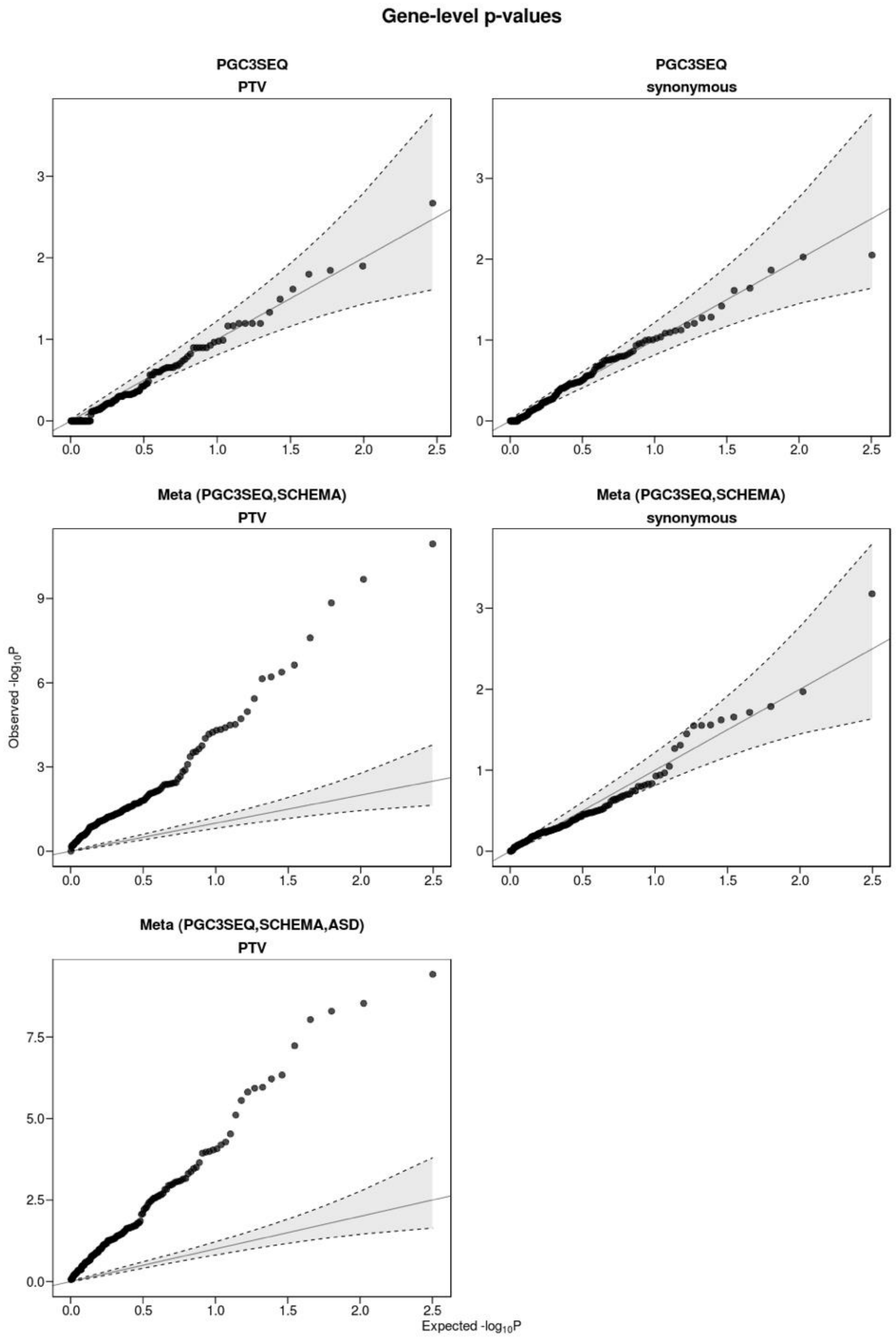

Supplementary Figure 10. *Q-Q* plot for gene-based tests of PTV (left) and synonymous variants (right). Observed $-\log_{10}$ *P*-values are plotted against expectation given a uniform distribution. Top: PGC3SEQ alone (n=11,580 cases and n=10,555 controls); Middle: Meta-analysis of PGC3SEQ and SCHEMA (n=35,828 cases and n=107,877 controls); Bottom: Meta-analysis of PGC3SEQ, SCHEMA, and the latest WES study of Autism Spectrum Disorder (ASD) (Satterstrom et al. 2020 summary statistics of synonymous variants were not available for ASD, n=47,814 cases and n=131,475 controls).

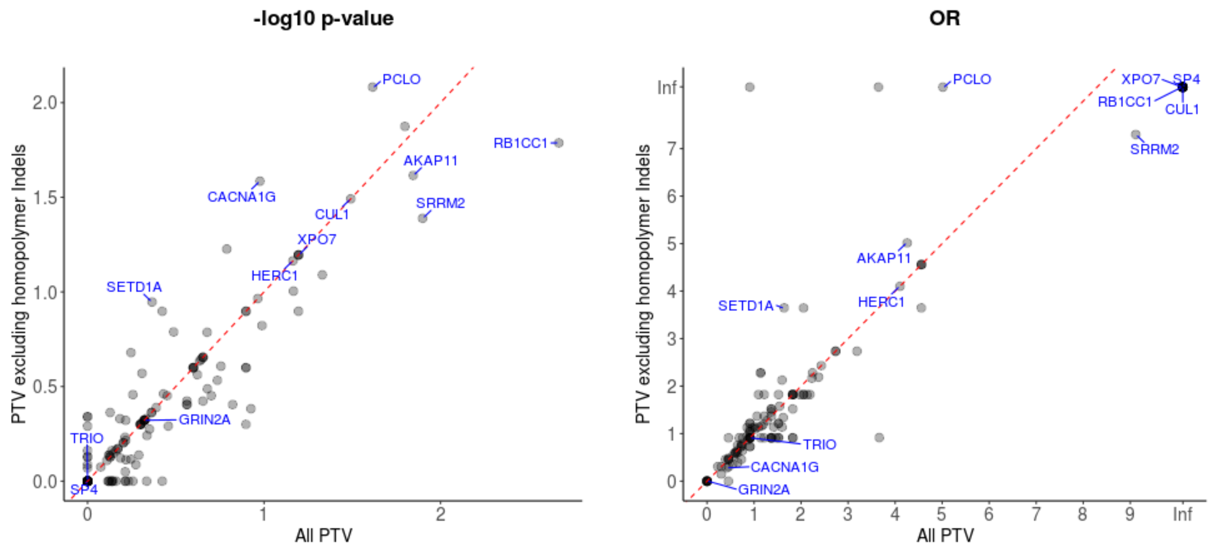

Supplementary Figure 11. Comparing gene-level burden p-values (right) and OR (left) for all PTVs (x axis) and for PTVs excluding Indels at homopolymer sites (y axis). Highlighted in blues are the nine genes implicated in SCHEMA and the three newly identified genes in PGC3SEQ.

Supplementary Tables

Supplementary Table10. Logistic regression for the global enrichment of rare variants, before and after excluding Indels.

	OR	CI lower	CI upper	P-value[a]
PTV	1.48	1.24	1.78	5.41E-06
PTV excluding homopolymer Indels	1.48	1.23	1.79	2.34E-05
PTV excluding all Indels	1.81	1.35	2.47	7.47E-05

[a] p-values are two-sided

Supplementary Table11. Gene-level burden test results for the twelve genes highlighted in Supplementary Figure 11

Gene	All PTVs				PTVs excluding homopolymer Indels			
	OR	p-value	count in cases	count in controls	OR	p-value	count in cases	count in controls
AKAP11	4.255	0.014	14	3	5.015	0.024	11	2
CACNA1G	0.421	0.105	6	13	0.280	0.026	4	13
CUL1	Inf	0.032	6	0	Inf	0.032	6	0
GRIN2A	0.000	0.477	0	1	0.000	0.477	0	1
HERC1	4.103	0.069	9	2	4.103	0.069	9	2
PCLO	5.015	0.024	11	2	Inf	0.008	8	0
RB1CC1	Inf	0.002	10	0	Inf	0.016	7	0
SETD1A	1.641	0.431	9	5	3.647	0.113	8	2
SP4	Inf	1.000	1	0	Inf	1.000	1	0
SRRM2	9.117	0.013	10	1	7.294	0.041	8	1
TRIO	0.911	1.000	3	3	0.911	1.000	3	3
XPO7	Inf	0.064	5	0	Inf	0.064	5	0

All p-values were obtained from Fisher's exact test, and are two-sided.
