# Supplementary material for: Schizophrenia risk conferred by rare protein-truncating variants is conserved across diverse human populations
Source: Nat Genet. 2023 Mar 13;55(3):369–76. doi: 10.1038/s41588-023-01305-1 (PMC10011128; doi:10.1038/s41588-023-01305-1)
Supplement: Supplementary file 2 — Reporting Summary [file 41588_2023_1305_MOESM2_ESM.pdf]

nature portfolio

Corresponding author(s): Dongjing Liu, Laura M. Huckins, Alexander W. Charney

Last updated by author(s): Nov 30, 2022

Reporting Summary

Nature Portfolio wishes to improve the reproducibility of the work that we publish. This form provides structure for consistency and transparency in reporting. For further information on Nature Portfolio policies, see our Editorial Policies and the Editorial Policy Checklist.

Statistics

For all statistical analyses, confirm that the following items are present in the figure legend, table legend, main text, or Methods section.

n/a	Confirmed	
☐	☒	The exact sample size (*n*) for each experimental group/condition, given as a discrete number and unit of measurement
☐	☒	A statement on whether measurements were taken from distinct samples or whether the same sample was measured repeatedly
☐	☒	The statistical test(s) used AND whether they are one- or two-sided<br>*Only common tests should be described solely by name; describe more complex techniques in the Methods section.*
☐	☒	A description of all covariates tested
☐	☒	A description of any assumptions or corrections, such as tests of normality and adjustment for multiple comparisons
☐	☒	A full description of the statistical parameters including central tendency (e.g. means) or other basic estimates (e.g. regression coefficient) AND variation (e.g. standard deviation) or associated estimates of uncertainty (e.g. confidence intervals)
☐	☒	For null hypothesis testing, the test statistic (e.g. *F*, *t*, *r*) with confidence intervals, effect sizes, degrees of freedom and *P* value noted<br>*Give P values as exact values whenever suitable.*
☒	☐	For Bayesian analysis, information on the choice of priors and Markov chain Monte Carlo settings
☐	☒	For hierarchical and complex designs, identification of the appropriate level for tests and full reporting of outcomes
☒	☐	Estimates of effect sizes (e.g. Cohen's *d*, Pearson's *r*), indicating how they were calculated

Our web collection on statistics for biologists contains articles on many of the points above.

Software and code

Policy information about availability of computer code

Data collection	No software and code was used in data collection.
Data analysis	Software and code used are described throughout the Supplementary Methods. In brief, we used Torrent Variant Caller version 5.8.0 to call variants from the raw sequence data. For QC and pre-processing, we used XGBoost v1.3 in Python v3.7.3, BCFtools v1.9, and PLINK v1.9. Re-analysis of the SCHEMA cohort was performed using Hail 0.1 and 0.2 (https://hail.is/). Main analyses in the PGC3SEQ data and its meta-analysis with SCHEMA were performed using R v3.6 with various libraries. Visualization was generated with ggplot2 v3.3.

For manuscripts utilizing custom algorithms or software that are central to the research but not yet described in published literature, software must be made available to editors and reviewers. We strongly encourage code deposition in a community repository (e.g. GitHub). See the Nature Portfolio guidelines for submitting code & software for further information.

Data

Policy information about availability of data

All manuscripts must include a data availability statement. This statement should provide the following information, where applicable:

- Accession codes, unique identifiers, or web links for publicly available datasets
- A description of any restrictions on data availability
- For clinical datasets or third party data, please ensure that the statement adheres to our policy

We describe all datasets in Online Methods and Supplementary tables/figures. The raw PGC3SEQ genotype and phenotype datasets are permitted to be distributed at the individual level and we have deposited the data in the database of Genotypes and Phenotypes dbGaP. The accession number is phs003138.v1.p1. We provide the aggregated variant counts at the gene and the gene-set level in supplementary tables. SCHEMA summary-level data is available as an online browser for viewing and download (https://schema.broadinstitute.org). SCHEMA individual-level whole-exome sequence data are hosted on and shared with the collaborating study

groups via the controlled-access Terra platform (https://app.terra.bio/). Requests for access to the controlled datasets are managed by data custodians of the SCHEMA consortium and the Broad Institute and are sent to sample contributing investigators for approval. The gnomAD database can be accessed at https://gnomad.broadinstitute.org.

Field-specific reporting

Please select the one below that is the best fit for your research. If you are not sure, read the appropriate sections before making your selection.

☒ Life sciences ☐ Behavioural & social sciences ☐ Ecological, evolutionary & environmental sciences

For a reference copy of the document with all sections, see nature.com/documents/nr-reporting-summary-flat.pdf

Life sciences study design

All studies must disclose on these points even when the disclosure is negative.

Sample size	Sample size was not predetermined in this study: we intended to aggregate samples from all available schizophrenia patient and control cohorts who have not been whole-exome or whole-genome sequenced at the time of study design. The sample size is sufficient because we were able to replicate findings from a previous study which had a bigger dataset, and meta-analyzed with that bigger study to further increase sample sizes.
Data exclusions	We describe sample ascertainment in detail in the Online Methods. We included only cases with a clear diagnosis of schizophrenia or schizoaffective disorders, and controls without a known diagnosis of a psychiatric disorder. We additionally described the criteria for which low-quality or related samples and low-quality variants were excluded in our study (see sections on Sample and Variant QC).
Replication	Our main analysis integrated case-control rare variant enrichment and gene discovery. We have access to the largest-to-date whole exome sequencing datasets of schizophrenia cohorts that are independent to our samples, and this dataset and our own dataset partially replicated one another. Some results reported in the other dataset were not replicated in our study.
Randomization	Case and control status of samples were assigned by investigators of contributing collections. We controlled for confounding factors (sequencing artifacts and population ancestry) by adjusting for those confounders in logistic regression.
Blinding	Blinding was not relevant to our study, as the genotype and phenotype data is determined/defined externally and could not be influenced by the analyst or during our aggregation steps.

Reporting for specific materials, systems and methods

We require information from authors about some types of materials, experimental systems and methods used in many studies. Here, indicate whether each material, system or method listed is relevant to your study. If you are not sure if a list item applies to your research, read the appropriate section before selecting a response.

Materials & experimental systems

n/a	Involved in the study	
☒	☐	Antibodies
☒	☐	Eukaryotic cell lines
☒	☐	Palaeontology and archaeology
☒	☐	Animals and other organisms
☐	☒	Human research participants
☒	☐	Clinical data
☒	☐	Dual use research of concern

Methods

n/a	Involved in the study	
☒	☐	ChIP-seq
☒	☐	Flow cytometry
☒	☐	MRI-based neuroimaging

Human research participants

Policy information about studies involving human research participants

Population characteristics	Supplementary Table S1 and S4 described contributing cohorts along with country of origin, the number of samples sequenced, and the number of samples retained in the final analysis. For each cohort, we give described description of the original recruitment and phenotypic ascertainment in Supplementary Information. To ensure compatibility with Psychiatric Genomics Consortium (PGC) definitions, we included samples with a diagnosis of schizophrenia and schizoaffective disorders in our analysis. The final dataset included 22,135 individuals from diverse ancestries, 40% of which are non-European (see Figure 1 for the number of subjects for each group). We do not have complete information on subjects' age and sex.
Recruitment	Patients were recruited originally as a part of numerous cohort studies, described in Supplementary Table S1 and Table S4. The ascertainment strategies of contributing cohorts are described in Supplementary Information.
Ethics oversight	The PGC3SEQ study protocol was approved by the Icahn School of Mount Sinai ethical review board (16-00101). The IRBs

Ethics oversight

that approved individual contributing studies are given in the Supplementary Note, Detail Cohort Description. Informed consent was obtained from all participants, and the institutional human subject review and ethics committees relevant to each contributing cohort approved the research.

Note that full information on the approval of the study protocol must also be provided in the manuscript.
